# Identifying a novel role for the master regulator *Tal1* in the Endothelial to Hematopoietic Transition

**DOI:** 10.1038/s41598-022-20906-0

**Published:** 2022-10-10

**Authors:** Yasmin Natalia Serina Secanechia, Isabelle Bergiers, Matt Rogon, Christian Arnold, Nicolas Descostes, Stephanie Le, Natalia López-Anguita, Kerstin Ganter, Chrysi Kapsali, Lea Bouilleau, Aaron Gut, Auguste Uzuotaite, Ayshan Aliyeva, Judith B. Zaugg, Christophe Lancrin

**Affiliations:** 1 European Molecular Biology Laboratory, EMBL Rome - Epigenetics and Neurobiology Unit, via E. Ramarini 32, 00015 Monterotondo, Italy; 2 European Molecular Biology Laboratory, Centre for Biomolecular Network Analysis, Meyerhofstrasse 1, 69117 Heidelberg, Germany; 3 European Molecular Biology Laboratory, Structural and Computational Biology Unit, Meyerhofstrasse 1, 69117 Heidelberg, Germany; 4 European Molecular Biology Laboratory, EMBL Rome - Epigenetics and Neurobiology Unit, Bioinformatics Services, via E. Ramarini 32, 00015 Monterotondo, Italy; 5 Present Address: Therapeutics Discovery, Pharmaceutical Companies of Johnson & Johnson, Janssen Research & Development, Turnhoutseweg 30, 2340 Beerse, Belgium; 6 Present Address: Max Planck Institute for Molecular Genetics, Ihnestraße 63-73, 14195 Berlin, Germany

**Keywords:** Differentiation, Haematopoiesis, Reprogramming, Stem cells, Transdifferentiation, Transcription, Transcriptomics, Embryonic stem cells, Haematopoietic stem cells, Multipotent stem cells, Pluripotent stem cells, Reprogramming, Stem-cell differentiation, Transdifferentiation, Developmental biology, Stem cells

## Abstract

Progress in the generation of Hematopoietic Stem and Progenitor Cells (HSPCs) in vitro and ex vivo has been built on the knowledge of developmental hematopoiesis, underscoring the importance of understanding this process. HSPCs emerge within the embryonic vasculature through an Endothelial-to-Hematopoietic Transition (EHT). The transcriptional regulator *Tal1* exerts essential functions in the earliest stages of blood development, but is considered dispensable for the EHT. Nevertheless, *Tal1* is expressed with its binding partner *Lmo2* and it homologous *Lyl1* in endothelial and transitioning cells at the time of EHT. Here, we investigated the function of these genes using a mouse embryonic-stem cell (mESC)-based differentiation system to model hematopoietic development. We showed for the first time that the expression of TAL1 in endothelial cells is crucial to ensure the efficiency of the EHT process and a sustained hematopoietic output. Our findings uncover an important function of *Tal1* during the EHT, thus filling the current gap in the knowledge of the role of this master gene throughout the whole process of hematopoietic development.

## Introduction

An important goal of regenerative medicine is the production of Hematopoietic Stem and Progenitor Cells (HSPCs) in vitro or ex vivo for clinical applications. Remarkable proofs-of-concept have been achieved in recent years exploiting current knowledge on the developmental processes and molecular regulators of developmental hematopoiesis^1,2^. This highlights the importance of gaining a deep understanding of this biological process to design effective regenerative approaches.

During embryonic life, HSPCs are produced transiently within the major embryonic arteries from specialized endothelial cells known as Hemogenic Endothelium (HE)^3,4^. This event is conserved across vertebrate species, and has been identified as a transdifferentiation termed “Endothelial to Hematopoietic Transition” (EHT)^5–7^.

The aim of our work was to assess the contribution of *Tal1*, *Lmo2* and *Lyl1* to the generation of pre-hematopoietic stem and progenitor cells (Pre-HSPCs), which represent the intermediate stage of the EHT^8^. *Tal1*, *Lmo2* and *Lyl1* form with *Runx1*, *Gata2*, *Cbfb*, *Erg* and *Fli1* a group of eight master regulators the combined activity of which regulates HSPC development^8–10^. Using a mouse embryonic-stem-cell (mESC)-based hematopoietic differentiation model, we have previously identified two opposing forces within these factors, which determine whether cells retain an endothelial identity (*Erg* and *Fli1*) or undergo EHT (*Runx1*, *Cbfb* and *Gata2*)^8^. The simultaneous co-expression of all eight factors maintains cells in the Pre-HSPC stage, but the exact role of *Tal1*, *Lmo2* and *Lyl1* in this context remained unclear^8^. *Tal1* is essential for the onset of hematopoiesis and is required for the generation of the HE, but its expression in endothelial cells is considered dispensable for the EHT to take place^3,11–16^. Indeed, the conditional ablation of *Tal1* in *Tie2*-expressing cells did not impair the initial stages of mouse hematopoiesis^15^ and its conditional re-expression in *Tal1*^*-/-*^ mESCs-derived cultures could not rescue hematopoiesis after endothelial cells were formed, which led to conclude that *Tal1* exerts its essential function before endothelial cell formation to prime the cells towards a hematopoietic fate, but does not have a prominent role at later stages^12^. In the first study, however, the lag between transcription of the *Cre*-recombinase and the actual downregulation of the TAL1 protein could have allowed enough time for HE cells and Pre-HSPCs to mature properly. Conversely, in the second study the lack of TAL1 should have impaired HE generation, likely explaining why its re-expression at later stages could not rescue hematopoietic cell formation.

LMO2 is a scaffold protein and a TAL1-binding partner, and its role in embryonic hematopoiesis mostly mirrors that of TAL1^17–19^. However, while *Tal1* ablation from mESCs completely abrogates hematopoiesis in vitro, *Lmo2* ablation is permissive to the generation of Pre-HSPCs and leads to later defects, suggesting that it is required for Pre-HSPC maturation^3,11,17^. LYL1 is a bHLH protein highly homologous to TAL1 in the bHLH domain^18,19^. Possibly owing to this, *Lyl1* was able to compensate for the loss of *Tal1* in adult HSCs^20^. However, it could not rescue hematopoiesis in vitro from *Tal1*^*-/-*^ mESCs^23^. The relevance of this protein for hematopoietic development is elusive, as *Lyl1*^-/-^ mice displayed only mild hematopoietic defects, while the generation of *Lyl1*^*-/-*^ mice with an 80% reduction in *Tal1* expression in the erythroid compartment revealed a functional redundancy between *Lyl1* and *Tal1* in primitive erythropoiesis^21^. *Tal1*, *Lmo2* and *Lyl1* are co-expressed at the single-cell level in Pre-HSPCs^8^ and *Tal1* overexpression in mouse yolk-sac endothelial cells increased their hematopoietic output, suggesting a role for it in the EHT^22^.

In the present work, combining gain-of-function experiments to multi-omics approaches in vitro we found that *Tal1*, *Lmo2* and *Lyl1* promote the activation of the hematopoietic program and appear to be involved in the transcriptional repression of VSM related genes. Remarkably, using a Tet-on system to control the expression of *Tal1* in our mESC-based hematopoietic differentiation system, we showed for the first time that TAL1 expression in the endothelium is important for the efficiency of the EHT and for a sustained hematopoietic output.

## Results

### The simultaneous overexpression of *Runx1*, *Cbfb*, *Gata2*, *Erg* and *Fli1*, but not that of *Tal1*, *Lmo2* and *Lyl1* generates a hemangioblast culture enriched in Pre-HPCs.

**Figure 1:**
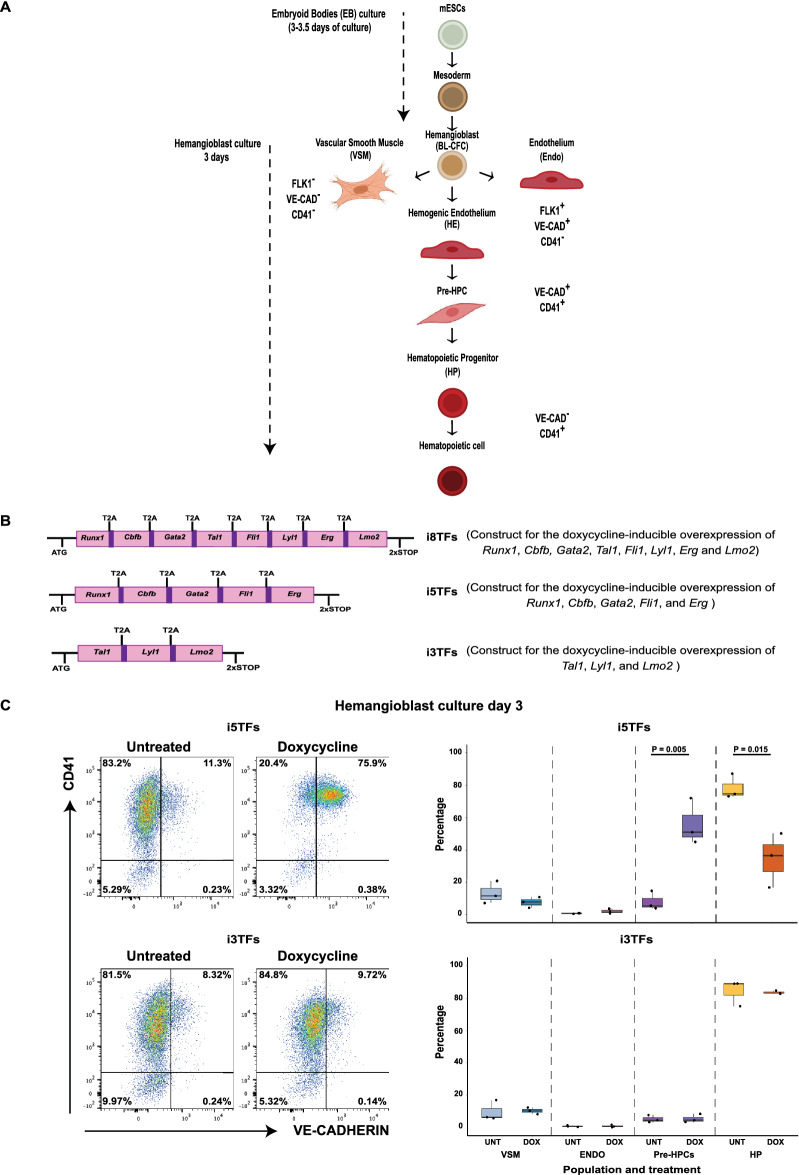
The simultaneous overexpression of *Runx1*, *Cbfb*, *Gata2*, *Erg* and *Fli1*, but not that of *Tal1*, *Lmo2* and *Lyl1* generates a hemangioblast culture enriched in cells resembling Pre-HPCs. (**A**) Scheme of the mESC differentiation system used in the study showing the cell-surface markers used to identify the different cell populations. (**B**) Schemes describing the transgenes used in the study. T2A = viral T2A peptide sequences inserted between the coding sequences of the genes to exploit the “ribosomal skipping” mechanism for the production of all proteins from one single transcript. (**C**) FACS analysis results showing the expression of VE-CAD and CD41 in day 3 hemangioblast cultures from i5TFs and i3TFs mESCs treated with doxycycline at day 1 of culture. Left: representative FACS plots. Right: Box plots summarizing the results of 3 experiments. Significance was determined by Analysis of Variance (ANOVA) test. Error bars correspond to standard deviations.

To investigate the role of *Tal1*, *Lmo2* and *Lyl1* in the EHT, we exploited a mESC-based differentiation system that models hematopoietic development *in vitro*^3^. The differentiation system and the cell-surface markers used throughout this study to identify and isolate the different cell populations in culture are described in Fig. 1A. In our previous work^8^ we had shown that the doxycycline (dox)-induced simultaneous overexpression of *Tal1*, *Lmo2* and *Lyl1* with *Erg*, *Fli1*, *Runx1*, *Cbfb* and *Gata2* (8TFs) (Fig. 1B) gave rise to mESC-derived hematopoietic cultures (i.e. “hemangioblast cultures”) highly enriched in the in vitro equivalents of Pre-HSPCs. Since they lack blood stem cell ability, these progenitors will be referred to as Pre-Hematopoietic Progenitor Cells (Pre-HPCs) hereafter. To identify the contribution of *Tal1*, *Lmo2* and *Lyl1* to the Pre-HPC phenotype, we generated two additional mESC lines using the inducible cassette exchange method previously described, which can overexpress *Tal1*, *Lmo2* and *Lyl1* alone (3TFs) and *Runx1*, *Cbfb*, *Gata2*, *Erg* and *Fli1* (5TFs) in a dox-inducible manner (Fig. 1B). Differentiation of the new cell lines confirmed that both were able to generate VSM (VE-CAD^-^CD41^-^), endothelial (Endo, VE-CAD^+^CD41^-^), Pre-HPCs (VE-CAD^+^CD41^+^) and hematopoietic progenitor cells (HP, VE-CAD^-^CD41^+^) after 3 days of hemangioblast culture, as assessed by morphological analysis and flow cytometry (Fig. 1C). The overexpression of the 3TFs and 5TFs was induced by treatment with dox at day 1 of hemangioblast culture, and the cultures were analyzed by flow cytometry two days after treatment. We found that just like the individual overexpression of the 3TFs^8^, their simultaneous overexpression did not have an obvious impact on the composition of the culture (Fig. 1C). In stark contrast, the overexpression of the 5TFs generated a culture highly enriched in Pre-HPCs (Fig. 1C). A FACS sort of the Pre-HPCs produced by induction of the 5TFs and the testing of their hematopoietic potential clearly showed their ability to generate a diverse set of blood cells in vitro (Supplementary Fig. 1). This is similar to the effect of the overexpression of the 8TFs^8^.

### The simultaneous overexpression of the 5TFs transdifferentiates sorted eVSM cells into VE-CAD^+^CD41^+^ cells less efficiently compared to the 8TFs

**Figure 2:**
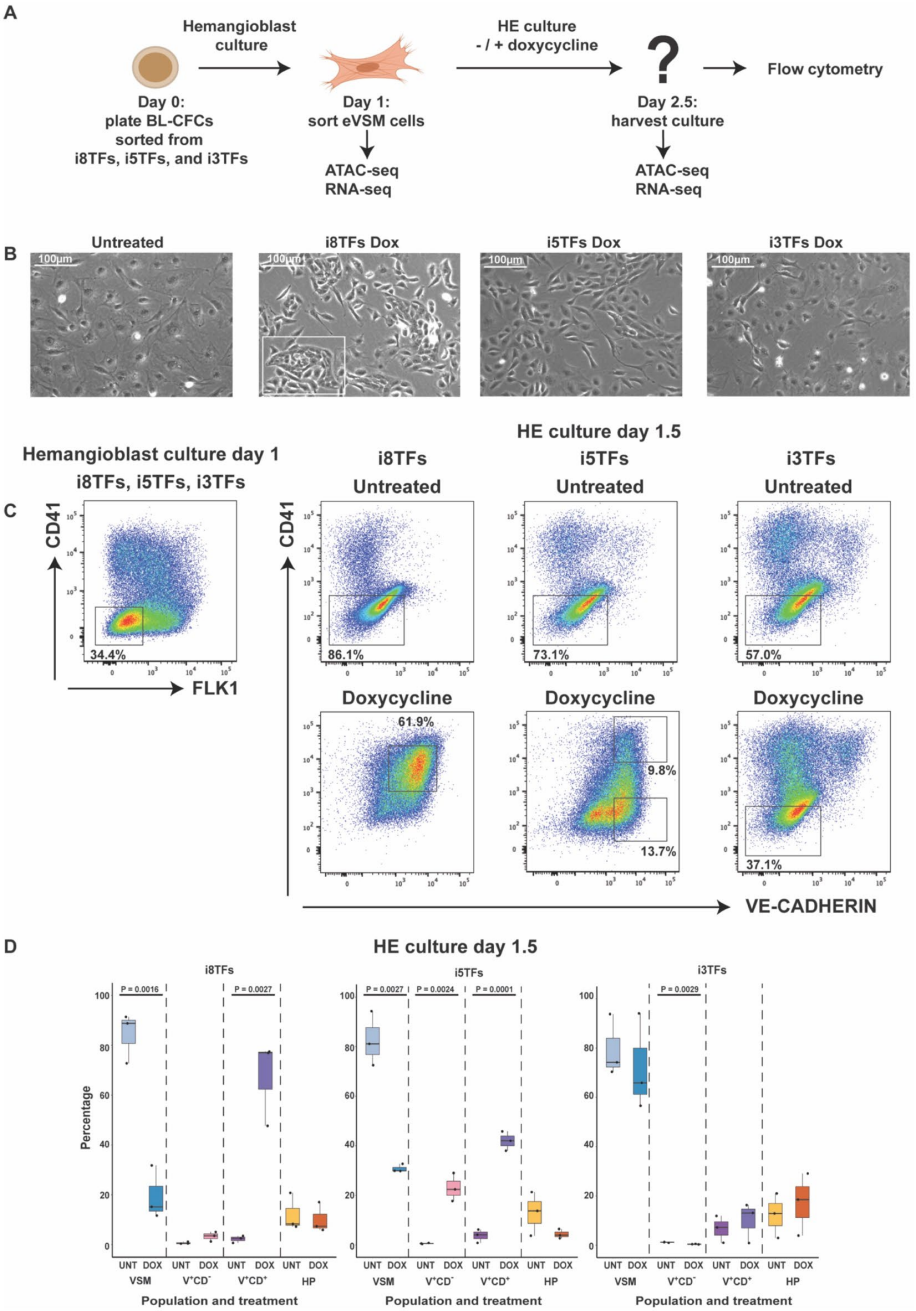
The simultaneous overexpression of the 5TFs transdifferentiates sorted eVSM cells into VE-CAD^+^CD41^+^ cells less efficiently compared to the 8TFs. (**A**) Experimental layout of the work. (**B**) Representative microscopy images of the indicated conditions at day 2.5 HE culture. VSM cells appear as large wide-spread cells; V^+^CD^−^ cells appear as elongated cells; HPs appear as round floating cells. The white box highlights an example of the endothelial cell cluster from which hematopoietic cells normally arise. (**C**) Flow cytometry analyses of the indicated conditions. The squares highlight the populations sorted for cell culture and molecular biology. (**D**) Box plots showing the relative frequencies of VSM, V^+^CD^−^, V^+^CD^+^, and HP populations in HE culture from i8TFs, i5TFs and i3TFs lines (n = 3). Significance was determined by Analysis of Variance (ANOVA) test. Error bars correspond to standard deviations.

Through careful examination of the effect of 5TFs overexpression in hemangioblast cultures, we noticed the presence of a residual VSM population (Fig. 1C) which was instead almost absent in cultures where the 8TFs were overexpressed^8^. As *Tal1* has been reported to act as a repressor of cardiac fate in mesodermal and endothelial cells^23–27^, this difference could reflect a role of the 3TFs also in repressing the VSM identity. On the other hand, the overexpression of the 3TFs alone did not lead to a reduction of VSM cell frequency, making it difficult to have a firm conclusion on the role of these 3TFs. We decided therefore to use an alternative assay to investigate the changes induced by differential transcription-factor overexpression. We chose a population enriched in VSM cells (eVSM) (Fig. 2), because they provide a non-hematopoietic and non-endothelial background against which to test the activity of transcription factors. Moreover, they can be transdifferentiated into VE-CAD^+^CD41^+^ cells that phenotypically resemble Pre-HPCs by the simultaneous overexpression of the 8TFs (Figs. 1A and 2C)^8^. As schematized in Fig. 2A, we differentiated mESCs from the i8TFs, i5TFs and i3TFs cell lines and FACS sorted from each a population of FLK1^-^CD41^-^ eVSM cells (parent eVSM) at day 1 of hemangioblast culture (Fig. 2A,C). Sorted FLK1^-^CD41^-^ cells were re-plated in a hemogenic endothelium (HE) medium that promotes hematopoiesis^3^ in the absence or presence of dox to induce the overexpression of the transcription factors (Fig. 2A). The cells were harvested after 1.5 days of HE culture and analyzed by flow cytometry (Fig. 2A–D). The overexpression of the 8TFs recapitulated previously published results, with the majority of the cells in culture co-expressing VE-CAD and CD41 (Fig. 2C,D). Differently from the hemangioblast culture, the overexpression of the 5TFs in sorted eVSM generated a culture enriched in VE-CAD^+^ cells, a large fraction of which did not co-express the hematopoietic cell-surface marker CD41 (Fig. 2C,D). Instead, similar to the hemangioblast culture, the overexpression of the 3TFs alone did not obviously alter the expression pattern of CD41 and VE-CAD in cultured cells compared to untreated controls, with the majority of the cells in culture displaying a VSM morphology (Fig. 2B–D). The overexpression of the 5TFs alone was therefore less efficient compared to that of all 8TFs in promoting the transdifferentiation of eVSM cells into VE-CAD^+^CD41^+^ cells. Thus, even though the 3TFs were not sufficient on their own to alter the phenotype of eVSM cells, they seemed to increase the ability of the 5TFs to promote the acquisition of a Pre-HPCs phenotype in non-hematopoietic cells.

### The endogenous *Tal1* and *Lmo2* genes were differentially expressed in VE-CAD^+^CD41^-^ and VE-CAD^+^CD41^+^ cells generated by 5TFs overexpression

**Figure 3:**
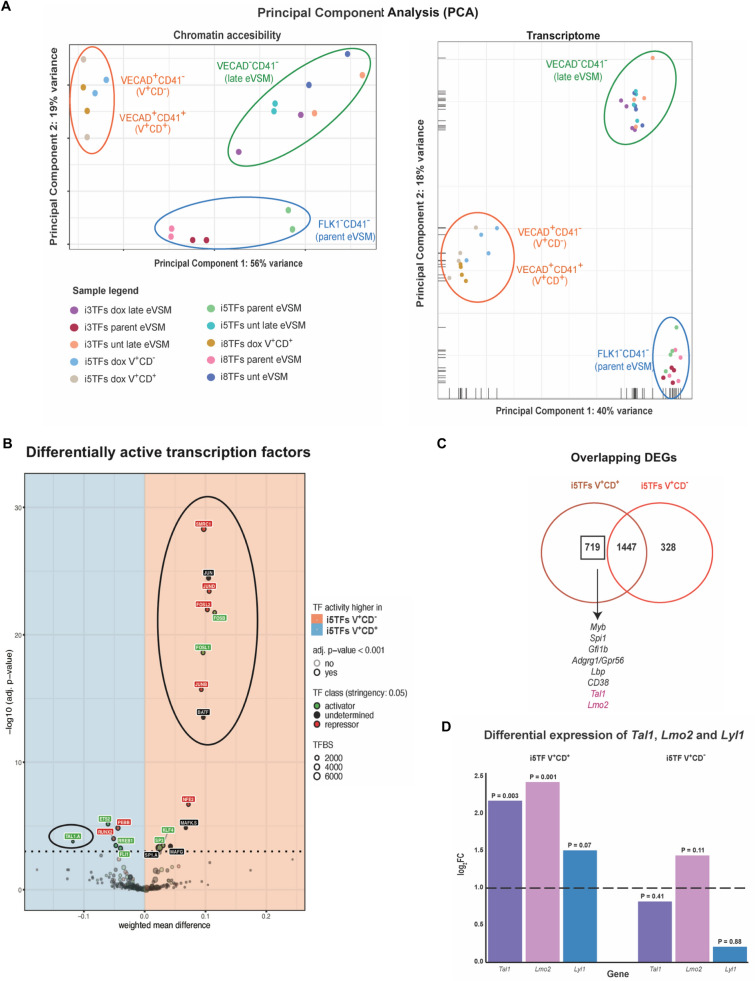
The endogenous *Tal1* and *Lmo2* genes were differentially expressed in i5TFs V^+^CD^+^ compared to i5TFs V^+^CD^−^ cells. (**A**) PCA plots of ATAC-seq results (n = 2) (left panel) and RNA-seq (n = 4) results (right panel). Parent eVSM = FLK1^−^CD41^−^ cells sorted at day 1 of hemangioblast culture; late eVSM = VE-CAD^−^CD41^−^ cells sorted at day 1.5 of HE culture; V^+^CD^−^ = VE-CAD^+^CD41^−^ cells sorted at day 1.5 of HE culture; V^+^CD^+^  = VE-CAD^+^CD41^+^ cells sorted at day 1.5 of HE culture; dox = cultured in HE medium with doxycycline; unt = cultured in HE medium without doxycycline. (**B**) Results of diffTF analysis for the indicated cell lines. TFs identified as more active in i5TFs V^+^CD^+^ are displayed in the blue quadrant, TFs identified as more active in i5TFs V^+^CD^-^ are displayed in the red quadrant. TFs classified as activators are labeled in green, TFs classified as repressors are labeled in red, differentially active TFs that couldn’t be clearly classified as either are labeled in black. 5% of TFs were classified as activators or repressors (TF class stringency: 0.05) based on the Pearson correlation index. The x axis (weighted mean difference) shows the difference in TF activity between the two conditions. The y axis displays the significance of the TFs. The significance threshold is indicated with a dotted line (FDR adjusted *p*-value < 0.05). TFs are indicated as a dot. The size of each dot is proportional to the number of predicted genomic TFBS for each (TFBS). (**C**) Venn diagrams comparing the DEGs in i5TFs V^+^CD^+^ and i5TFs V^+^CD^-^ . A subset of hematopoietic genes differentially expressed in V^+^CD^+^ but not V^+^CD^-^ cells is shown. (**D**) Bar plots showing the differential expression (log2FC) of *Tal1*, *Lmo2* and *Lyl1* in i5TFs V^+^CD^+^ and i5TFs V^+^CD^−^ cells compared to untreated eVSM cells, identified by RNA-seq.

To get a mechanistic insight on the changes induced by differential transcription factor overexpression in eVSM cells, we FACS sorted the most abundant cell types that were generated in the three dox-treated conditions as well as VE-CAD^-^CD41^-^ eVSM (late eVSM) cells from untreated controls, and assessed their chromatin accessibility and transcriptional profile by ATAC-seq and RNA-seq (Fig. 2A and C)^28,29^. The sorting gates are shown in Fig. 2C. Principal component analysis (PCA) on the chromatin accessibility and transcriptional data (Fig. 3A) confirmed that the parent eVSM sorted from the three cell lines were qualitatively similar, as they clustered together in both analyses. Cells in which the 8TFs and 5TFs were overexpressed greatly differed from untreated controls in their chromatin accessibility and transcriptional landscapes, while cells where the 3TFs were overexpressed appeared to be overall similar to untreated controls, in line with the morphological and cytofluorimetric observations.

The cells generated by the induction of the 5TFs in eVSM cells differed in the expression of the hematopoietic cell-surface marker CD41 (Fig. 2C,D), suggesting that the hematopoietic program was differentially active in these cells. To get more insights into the differences at the molecular level between these cells we decided to exploit the bioinformatic tool diffTF^30^. Based on the ATAC-seq data and a set of predicted or validated TF binding sites, diffTF estimates which transcription factors are differentially active between two conditions (hereafter: i5TFs V^+^CD^−^ (i.e. endothelial-like VE-CAD^+^CD41^−^ cells) versus i5TFs V^+^CD^+^ (i.e. Pre-HPC-like VE-CAD^+^CD41^+^ cells) (Fig. 3B).

By applying diffTF to our data, we identified a number of transcription factors that appeared to be differentially active in i5TFs V^+^CD^-^ versus i5TFs V^+^CD^+^ (Fig. 3B). Some of the most active transcription factors in i5TFs V^+^CD^−^ compared to i5TFs V^+^CD^+^ were specific to VSM identity such as the SWI/SNF subunit *Smrc1*/*Smarcc1* and the Activator Protein-1 (AP-1) transcription factors *Fosl1*, *Fosl2*, *Jun, Junb, Jund* and *Batf* (Fig. 3B). Indeed, both families of proteins have known roles in the development and physiology of muscle cells, and the activity of these transcription factors was consistently found to characterize untreated eVSM cells in our diffTF analysis (Supplementary Fig. 2)^31–34^. In contrast, one of the most active transcription factors in i5TFs V^+^CD^+^ compared to i5TFs V^+^CD^-^ was *Tal1* (Fig. 3B). This unexpected finding was reinforced by the fact that *Tal1* was upregulated in i5TFs V^+^CD^+^ but not in i5TFs V^+^CD^−^ relative to i5TFs VSM (Fig. 3C,D). *Lmo2* followed the same expression pattern (Fig. 3C,D). This finding suggests that the endogenous expression of *Tal1* and *Lmo2* may have a role in the generation of VE-CAD^+^CD41^+^ cells following the overexpression of the 5TFs.

### 5TFs overexpression in hemangioblast cultures cannot generate VE-CAD^+^CD41^+^ cells in the absence of a functional TAL1

**Figure 4:**
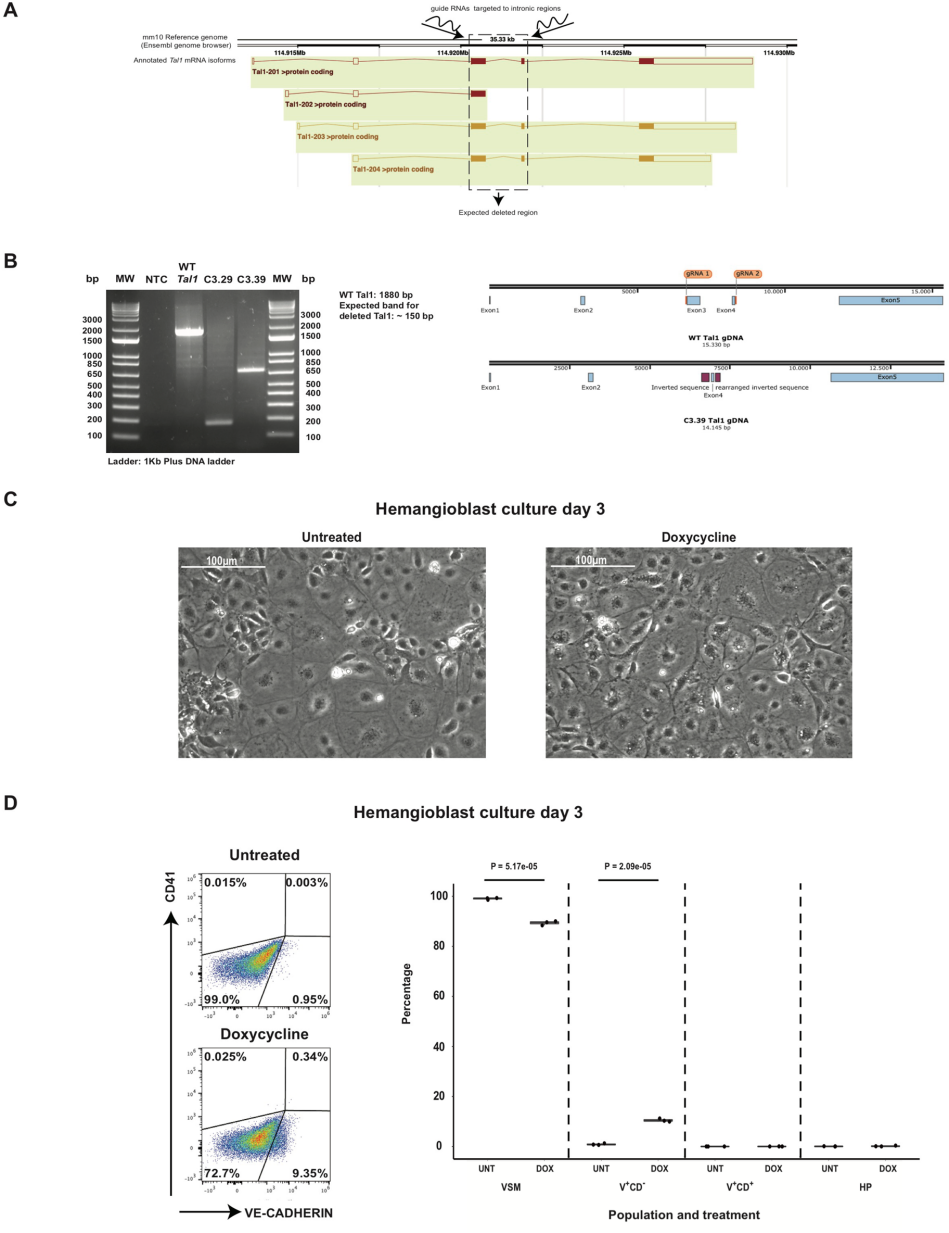
5TFs overexpression in hemangioblast cultures cannot generate VE-CAD^+^CD41^+^ cells in the absence of a functional TAL1*.* (**A**) CRISPR/Cas9 strategy used to disrupt the *Tal1* gene in the i5TFs mESC line (figure downloaded and modified from the Ensembl genome browser). (**B**) Left: Electrophoresis showing the genotyping of the *Tal1* mutant mESCs clones used for experiments. Right: Scheme of the WT *Tal1* gene and the *Tal1* gene from mutant clone C3.39 reconstructed based on the sequencing results. (**C**) Representative microscopy images of the indicated conditions at day 3 of hemangioblast culture. (**D**) Representative flow cytometry analyses of the indicated conditions (left panels) and box plots showing the relative frequencies of VSM, V^+^CD^−^, V^+^CD^+^ and HP populations at day 3 of hemangioblast culture (n = 3). Significance was determined by Analysis of Variance (ANOVA) test. Error bars correspond to standard deviations.

Following the previous analysis, we hypothesized that the loss of *Tal1* would prevent the formation of VE-CAD^+^CD41^+^ cells after the overexpression of the 5TFs. We focused on *Tal1* because it has been shown that the *Lmo2* ablation from mESCs allows the generation of bona-fide Pre-HPCs, albeit at lower frequency compared to *Lmo2*^+*/*+^ mESCs, and *Lyl1* was not significantly upregulated in our datasets (Fig. 3D)^17^. To test our hypothesis, we decided to generate a *Tal1* knock-out in the i5TFs line genetic background. Using the CRISPR/CAS9 technology, we obtained two mutant lines (Fig. 4A,B, Supplementary Fig. 3). Differentiating these lines, we verified that the deletion in the *Tal1* gene prevented the formation of blood and endothelial cells, as only VSM cells were generated (Fig. 4C,D). This confirmed the absence of a functional TAL1^3^. Following the overexpression of the 5TFs in these cultures, we could only produce cells expressing the endothelial marker VE-CAD. No cells expressing CD41 were detected (Fig. 4C,D). Thus, we concluded that a functional TAL1 is required for the 5 other TFs to generate VE-CAD^+^CD41^+^ cells from VSM.

### Hierarchical clustering of differentially-expressed genes identified functional interactions between the 3TFs and the 5TFs on transcriptional regulation

**Figure 5:**
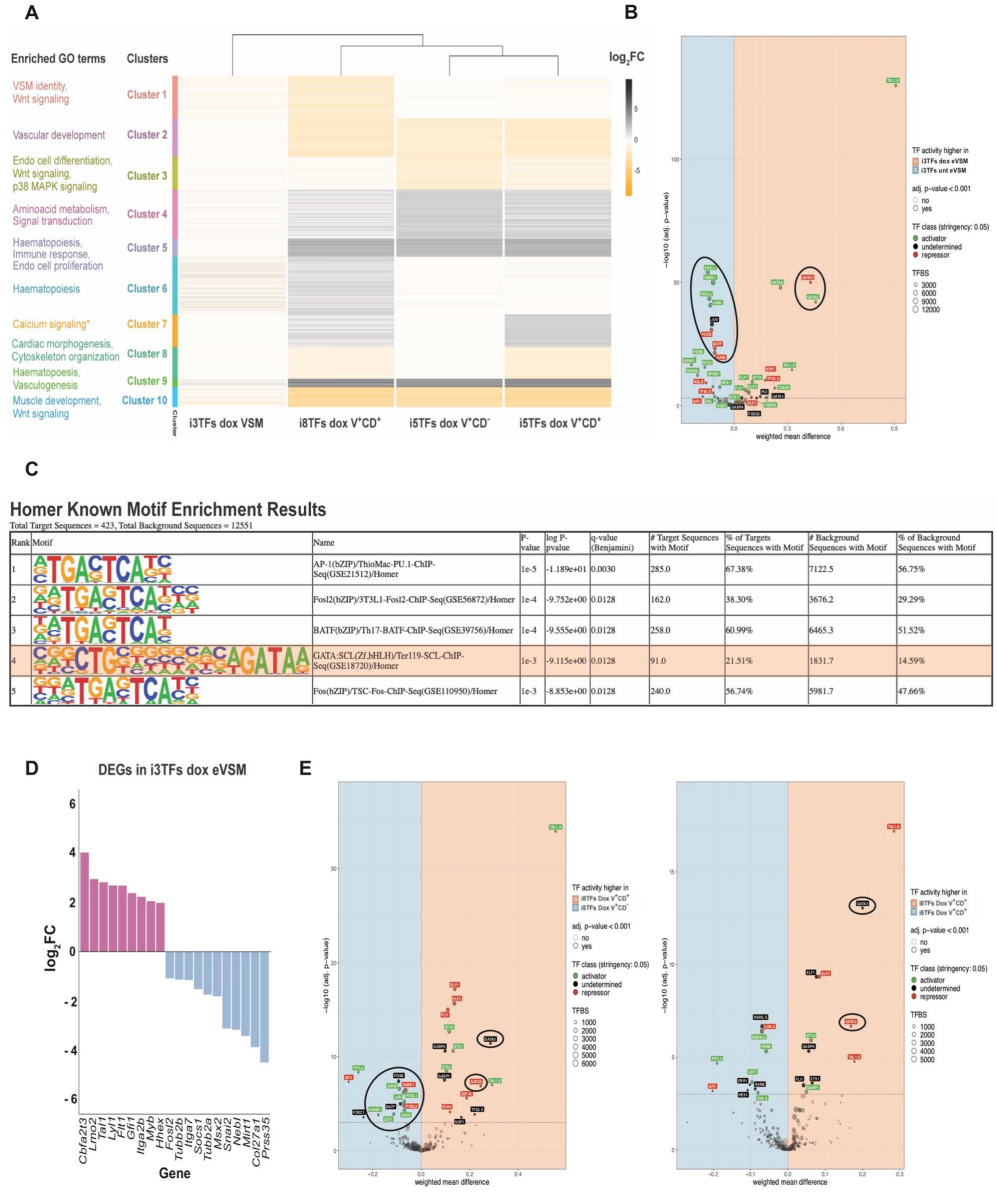
3TFs overexpression contributes to the silencing of the muscle transcriptional program and the activation of the hematopoietic one in eVSM. (**A**) Heatmap showing the expression of genes from the clusters indicated in the figures. The most representative GO terms enriched in each cluster are shown in the left. * KEGG enrichment (no significant GO terms could be found for cluster 7). (**B**) diffTF volcano plot comparing i3TFs untreated (unt) eVSM to i3TFs dox-treated (dox) eVSM. A detailed explanation of the plot can be found in Fig. 3. TFs characteristic of VSM cells and hematopoietic TFs are circled. (**C**) Results of the “Known Motif Enrichment” analysis performed with the HOMER software on the differentially expressed genes in i3TFs dox eVSM. The top 5 enriched transcription-factor motifs are shown. The Tal1 motif is highlighted in orange. (**D**) Selection of differentially expressed genes (DEGs) comparing i3TFs unt eVSM to i3TFs dox eVSM. Hematopoietic genes are shown in pink, VSM-related genes are shown in blue. (**E**) diffTF volcano plots comparing i8TFs V^+^CD^+^ to i5TFs V^+^CD^+^ (left panel) and i8TFs dox V^+^CD^+^ to i5TFs dox V^+^CD^−^ (right panel). A detailed explanation of the plots can be found in Fig. 3. VSM and hematopoietic TFs are circled.

Using the DESEq2 package^35^ we identified the differentially expressed genes (DEGs) between untreated controls and cells overexpressing the 8TFs, the 5TFs and the 3TFs (Supplementary file S1). To compare the effect of differential transcription factor overexpression we performed a hierarchical clustering analysis on the DEGs of the four sorted populations, dividing our dataset into 10 clusters containing genes with a similar expression pattern (Fig. 5A, Supplementary file S3). The clusters that were generated seem to reflect the relative contribution of the 5TFs and the 3TFs to the transcriptional changes induced by the 8TFs, and the acquisition of a Pre-HPC-like identity. To exemplify this, cluster 1 contained mostly genes that were downregulated only in i8TFs V^+^CD^+^, suggesting that they were downregulated as a consequence of the combined action of the 5TFs with the 3TFs, while cluster 2 contained mostly genes that were clearly downregulated as a consequence of 5TF overexpression, with seemingly no contribution from the 3TFs. Such hierarchical clustering provided multiple pieces of evidence suggesting that a functional cooperation occurred among the 8TFs in regulating gene expression in i8TFs V^+^CD^+^. Indeed, we observed that subsets of genes were silenced (in cluster 1) and upregulated (in cluster 6) specifically when all 8TFs were overexpressed simultaneously, other genes were strongly silenced (in cluster 3) specifically when only the 5TFs but not the 8TFs were overexpressed, and a few genes (in clusters 6 and 10) appeared to be differentially regulated when the 3TFs were overexpressed alone or in combination with the other 5TFs. The hierarchical clustering analysis also highlighted transcriptional differences between the i5TFs V^+^CD^+^ and the i8TFs V^+^CD^+^ cells. This might suggest that the expression of the 3TFs has non-redundant roles in the transcriptional regulation of specific subsets of genes in Pre-HPCs.

### 3TFs overexpression likely contributes to the silencing of the muscle transcriptional program and the activation of the hematopoietic one in eVSM

Next, we performed GO analysis on the genes from each cluster (Fig. 5A, Supplementary Fig. 4–8, Supplementary file S4). This analysis allowed us to identify a contribution of the 3TFs to the downregulation of non-hematopoietic transcriptional programs, particularly the muscle transcriptional program (clusters 1 and 10), the upregulation of the hematopoietic one (clusters 6 and 9), as well as to the regulation of the Wnt, p38 Map kinase and BMP signaling pathways (clusters 3, 10). Indeed, we found cluster 1 to be enriched in GO terms related to functions that are important for muscle cells, such as extracellular matrix organization and muscle tissue development, as well as genes belonging to the Wnt signaling pathway (Fig. 5A, Supplementary Fig. 4A), while cluster 6 was predominantly enriched in GO terms related to hematopoiesis (Fig. 5A, Supplementary Fig. 4B).

In agreement with the GO analysis, we found with our diffTF analysis combined with the use of STRING (Fig. 5B & Supplementary Fig. 9) that the smooth muscle specific TFs were more active in i3TFs unt eVSM compared to i3TFs dox eVSM. In contrast, several hematopoietic TFs were more active in the dox-treated conditions. Moreover, we found among the genes that were upregulated in i3TFs dox eVSM cells important hematopoietic regulators such as *Cbfa2t3*^23^, the *Tal1* target *Gfi1*^10,36^ and *Myb*^37^ as well as the earliest murine hematopoietic marker *Itga2b*^38^ (Fig. 5D), indicating that 3TFs overexpression alone was likely able to promote a partial activation of the hematopoietic transcriptional program. Likewise, among the downregulated genes we found ones with reported roles in vascular smooth muscle (*Mirt1* and *Msx2*)^39,40^, in myogenesis (*Snai2* and *Socs1*)^41,42^, in directing hemangioblast cells towards the acquisition of a smooth muscle fate (*Fosl2*)^33^, cytoskeleton remodeling proteins and extracellular matrix components, suggesting that 3TFs overexpression alone was able to partially silence the muscle transcriptional program (Fig. 5D). Of note, nearly half of the DEGs in i3TFs dox eVSM cells were not in common with the DEGs in i8TFs V^+^CD^+^ (Supplementary Fig. 10), further supporting a functional interaction among the 8TFs that directs the transcriptional activity of these factors. A motif enrichment analysis with the HOMER software revealed an enrichment for the TAL1 (SCL) binding site, as well as binding sites for VSM transcription factors (e.g. AP-1, FOSL2), around the transcription starting site of DEGs upon 3TFs overexpression (Fig. 5C), supporting the results of our diffTF analysis (Fig. 5B).

To gain further insights on the 3TFs role, we analyzed the diffTF results comparing the i5TFs line to the i8TFs one (Fig. 5E). When comparing the i8TFs V^+^CD^+^ to the i5TFs V^+^CD^-^, the results appeared similar to the comparison between i5TFs V^+^CD^+^ and the i5TFs V^+^CD^-^ shown in Fig. 3B, with VSM TFs being the most active in i5TFs V^+^CD^-^ and hematopoietic TFs being the most active in i8TFs V^+^CD^+^. When we compared the i8TFs V^+^CD^+^ to the i5TFs V^+^CD^+^, however, we found that VSM TFs were no longer the most differentially active ones (Fig. 5E). Since the endogenous *Tal1* and *Lmo2* genes were upregulated in i5TFs V^+^CD^+^, this analysis provided orthogonal evidence suggesting that the upregulation of at least *Tal1* and *Lmo2* was involved in the downregulation of the VSM transcriptional program in the cells in which they were expressed.

### Rescue of *Tal1* expression during hemogenic endothelium culture restores the formation of VE-CAD^+^ CD41^+^ and VE-CAD^−^ CD41^+^ cells from endothelium and ensures a sustained hematopoietic output

**Figure 6:**
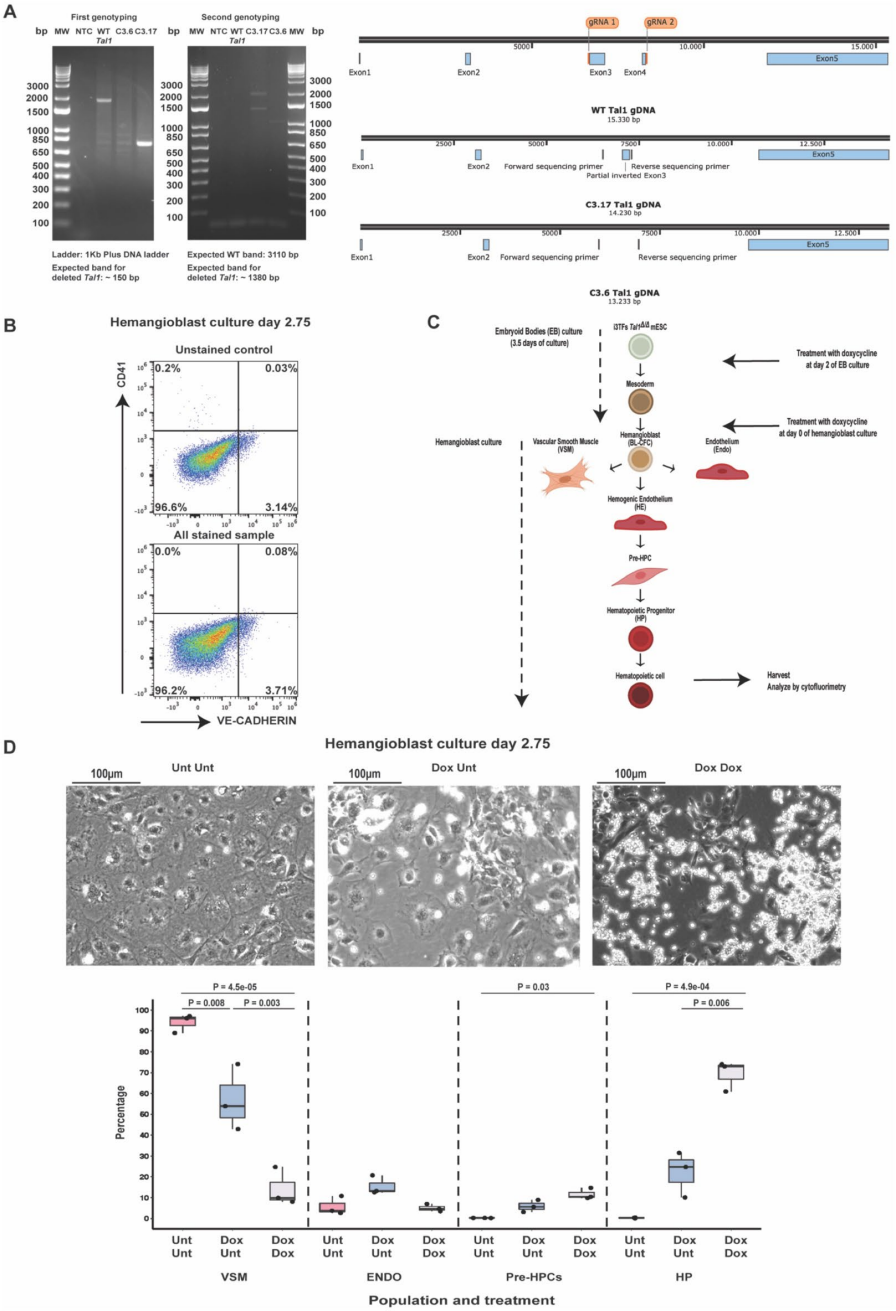
Characterization of the *Tal1 *^*Δ/Δ*^ inducible 3TFs embryonic stem cell line. (**A**) Left: electrophoresis showing the genotyping of the *Tal1* mutant mESCs clones used for experiments. Right: scheme of the WT *Tal1* gene and the *Tal1* gene from mutant clones C3.17 (allele with the shorter deletion) and C3.6 reconstructed based on the sequencing results. (**B**) Representative flow cytometry analyses of untreated day 1.75 hemangioblast cultures comparing the profile of unstained cells (top) and cultures stained for VE-CAD and CD41 (bottom). 3–4% of the cells in hemangioblast cultures were auto-fluorescent in the red (VE-CAD) channel. (**C**) Experimental layout used to characterize the mutant line. (**D**) Top: representative microscopic images of the indicated conditions. VSM cells appear as large wide-spread cells; Endo cells appear as elongated cells; HPs appear as round floating cells. Unt Unt = untreated; Dox Unt = doxycycline added at EB day 2; Dox Dox = doxycycline added at EB day 2 and at day 0 of hemangioblast culture. Bottom: box plots showing the relative frequencies of VSM, Endo, Pre-HPC and HP populations of HE culture from the indicated conditions (n = 3). Significance was determined by Analysis of Variance (ANOVA) test followed by Tukey HSD test for multiple test correction. Error bars correspond to standard deviations.

**Figure 7:**
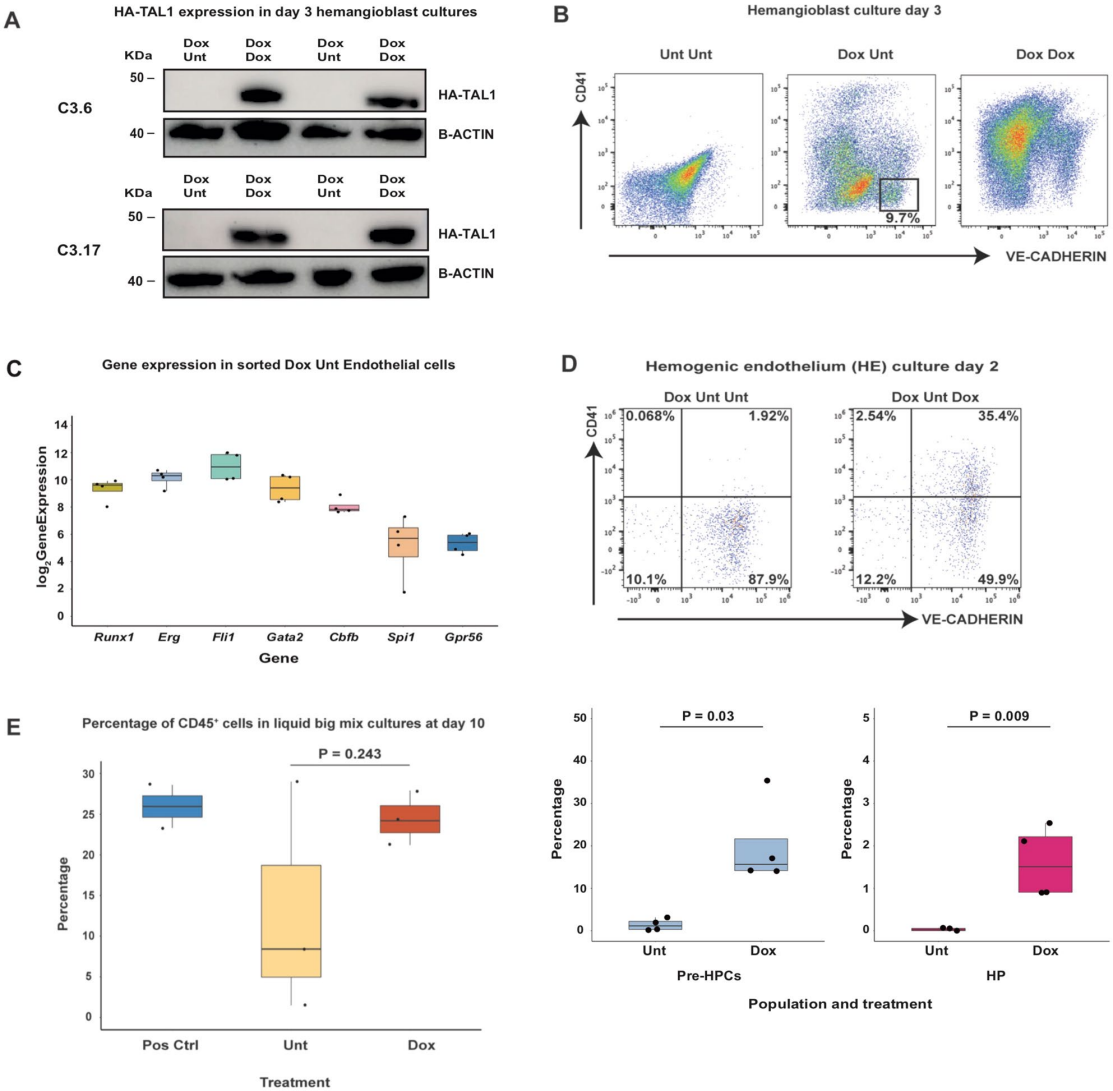
Rescue of *Tal1* expression during hemogenic endothelium culture allows the formation of Pre-HPCs and HP from endothelium. (**A**) Western-blot (WB) analysis showing the expression of transgenic HA-TAL1 in day 3 Dox Unt and Dox Dox i3TFs *Tal1*^*Δ/Δ*^ hemangioblast cultures. The WB was performed on two independent repeats for each cell line. B-ACTIN was used as a loading control. (**B**) Representative flow cytometry analysis of i3TFs *Tal1*^*Δ/Δ*^ Unt Unt, Dox Unt and Dox Dox hemangioblast cultures at day 3. The square highlights the endothelial cell population (VE-CAD^+^CD41^−^) sorted from the Dox Unt condition for subsequent hemogenic endothelium culture and RNA-seq. (**C**) RNA-seq (n = 4) analysis showing the expression of the indicated genes in the FACS sorted endothelial cell population. (**D**) Results of hemogenic endothelium cultures. Top: panels showing one representative flow cytometry analysis. Bottom: box plots summarizing four independent experiments. Significance was determined by Analysis of Variance (ANOVA) test. Error bars correspond to standard deviations. (**E**) Box plots summarizing the expression of CD45 in untreated and dox-treated HE cultures subjected to a liquid hematopoietic cell growth assay in the absence of doxycycline for 10 days. Whole i3TFs *Tal1*^***Δ/Δ***^ Dox Dox hemangioblast cultures were used as a positive control. Significance was determined by Analysis of Variance (ANOVA) test. Error bars correspond to standard deviations. Unt Unt = untreated; Dox Unt = doxycycline added at EB day 2; Dox Dox = doxycycline added at EB day 2 and at day 0 of hemangioblast culture; CD45 = pan-hematopoietic marker.

Our previous analyses prompted us to assess whether *Tal1* played a role in the formation of Pre-HPCs from the endothelium. The other studies that addressed this question did not find evidence to support a role for *Tal1* at this stage^12,15^. To address this question, we developed a new system. The key was to be able to ensure the activity of TAL1 in the mesoderm in order to produce endothelium and hemogenic endothelium, but to switch off its activity in the latter cell types in order to verify whether blood cell formation could progress. To make this new tool, we disabled the *Tal1* gene in the i3TFs line using the CRISPR/CAS9 method (Fig. 6A,B). As expected, no endothelial cells nor blood cells could be generated from these cell lines, and the expression of key hematopoietic transcription factors was drastically reduced (Supplementary Fig. 11), confirming the absence of a functional TAL1 (Fig. 6B,D). To perform our experiments, we induced *Tal1*, *Lmo2* and *Lyl1* expression by addition of dox at day 2 of EB differentiation (Fig. 6C), to produce FLK1^+^ mesodermal cells with hematopoietic potential. After sorting of FLK1^+^ cells, they were either plated with dox to maintain the expression of the 3TFs, or without to create a hemangioblast culture lacking the expression of a functional TAL1. Cells which were never exposed to dox (Unt Unt condition) were used as negative control. They were unable to produce any blood or endothelial cells but generated VSM as expected from cells lacking a functional TAL1. The constant treatment with dox (Dox Dox condition) led to a high frequency of VE-CAD^−^CD41^+^ cells **(**HP) after 2.75 days of culture. VE-CAD^+^CD41^−^ cells **(**Endo) and VE-CAD^+^CD41^+^ cells **(**Pre-HPCs) could also be detected. In contrast, in the condition in which dox was only added at the EB stage (Dox Unt condition), the frequency of VE-CAD^−^CD41^+^ cells was about three times lower than in the Dox Dox condition (Fig. 6D). Hematopoiesis was therefore still ongoing after removal of dox albeit at a lower efficiency. At day 3 of culture, transgenic TAL1 protein expression was undetectable in Dox Unt compared to the Dox Dox (Fig. 7A & Supplementary Fig. 12). It is at this stage that we decided to isolate endothelial cells from Dox Unt condition for hemogenic endothelium culture.

We isolated VE-CAD^+^CD41^−^ cells by FACS sorting from Dox Unt hemangioblast cultures at day 3 (Fig. 7B). Parts of these cells were assessed by RNA-seq while the rest was used for the in vitro culture assay. We found that these cells strongly expressed *Runx1*, *Erg*, *Fli1*, *Gata2* and *Cbfb*, as well as additional hematopoietic genes such as *Gpr56*, which is required for HSPC generation^43^, and *Spi1*, a regulator of HSPC differentiation (Fig. 7C, Supplementary file S5). The hemogenic endothelium culture was performed in absence or presence of dox. When the 3TFs were not induced, therefore in absence of transgenic TAL1 expression, none or very few CD41^+^ cells were detected. In contrast, dox addition led to a significant increase of CD41^+^ cell frequency. Most of these cells were VE-CAD^+^CD41^+^ (Pre-HPCs), but more mature VE-CAD^−^CD41^+^ (HP) were also found (Fig. 7D). Hematopoietic cell growth assay was performed following the hemogenic cell culture. We could consistently detect CD45^+^ cells from the culture in dox condition, while in untreated cultures the frequency of CD45^+^ cells was surprisingly variable, ranging from undetectable to levels comparable to those of dox-treated cultures (Fig. 7E & Supplementary Fig. 13). Taken together, these evidences seem to suggest that in the absence of a functional TAL1 the EHT process is highly inefficient, with a consequently inconsistent level of hematopoietic production (Fig. 7D). *Tal1* appears therefore to play an essential role in ensuring the efficient production of VE-CAD^+^CD41^+^ cells from the endothelium and a sustained hematopoietic output.

## Discussion

The aim of our study was to elucidate the function of the hematopoietic regulators *Tal1*, *Lmo2* and *Lyl1* during the Endothelial to Hematopoietic Transition (EHT). As their enforced expression in combination with the hematopoietic regulators *Runx1*, *Cbfb*, *Gata2*, *Erg* and *Fli1* was able to generate VE-CAD^+^CD41^**+**^ cells similar to Pre-HSPCs from mESC-derived hemangioblast cultures and mESC-derived VSM cells with very high efficiency^8^, we addressed our question by investigating their contribution to VE-CAD^+^CD41^+^ cell production in these systems. We compared the effect of overexpressing just *Tal1*, *Lmo2* and *Lyl1* (3TFs) and just *Runx1*, *Cbfb*, *Gata2*, *Erg* and *Fli1* (5TFs) to the overexpression of all eight factors (8TFs) together, at the phenotypic and molecular level.

The overexpression of the 3TFs alone appeared to be involved in the activation of the hematopoietic transcriptional program and the downregulation of muscle-related genes in eVSM. This was corroborated by our comparative analysis of the transcriptional changes induced by differential transcription factor overexpression. *Tal1* is essential for the hematopoietic commitment of mesodermal precursors^3,11^, and multiple evidences showed that it has an important role in suppressing the alternative cardiac and paraxial lineage fates^23–27^. However, a controversy still exists in the field on this matter, resulting from a sc-RNA-seq-based study on differentiating FLK1^+^ mesodermal cells from gastrulating embryos^44^. Here, we have provided the first experimental evidence to suggest that *Tal1*, in combination with its binding partner *Lmo2* and its homologous *Lyl1,* could help promote the silencing of the VSM transcriptional program. This effect is likely to be mediated for the most part by *Tal1* in partnership with *Lmo2,* as their endogenous upregulation in i5TFs V^+^CD^+^ appeared sufficient to down-modulate the activity of VSM transcriptional regulators according to our diffTF analysis. Our results are aligned with previous ones supporting a role for *Tal1* in the transcriptional repression of alternative fates. Nonetheless, more work is to be done to show that this effect is directly connected to the binding of these transcription factors to the VSM related genes. Definitive evidence would require chromatin occupancy analysis of the three transcription factors in our experimental system. It would be an interesting line of investigation to pursue in the future.

Our main finding is that TAL1 expression in endothelial cells is important to ensure the efficient production of VE-CAD^+^CD41^+^ cells and a sustained hematopoietic output (Fig. 7D) in our in vitro model. In their study that addressed this question in vivo, Schlaeger et al.^15^ found no evidence that *Tal1* has a role in the EHT. However, their experimental system might have allowed enough expression of *Tal1* before its excision for HE to mature and undergo EHT, something which was not excluded by the authors themselves when they concluded that “it remains to be address whether SCL plays more prominent roles in intra-embryonic endothelial cells before Tie2-Cre mediated excision becomes effective”. In line with this, our study has refined their findings by investigating in a more controlled manner the effect of TAL1 removal in the window of time between HE formation and the EHT, and this could explain the apparent discrepancy between our two studies. Additionally, our most recent findings showed that the few CD41^+^ cells that could be formed in the absence of TAL1 in HE cultures were able to give rise to CD45^+^ cells. It is therefore possible that a similar effect occurred also in vivo, allowing the survival of mice past E11.5 and preventing the identification of a defect in EHT, since the presence and composition of intra-aortic hematopoietic clusters (IAHC) was not assessed in *Tal1*^*fl/fl*^ Tie2-CRE mice.

Zhen F et al.^45^ investigated two different Tal1 isoforms in the zebrafish embryo and identified their temporal requirement, showing that β-Tal1 is required for the specification of the HE and α-Tal1 is required later for the maintenance of HSCs. The study did not identify however a role of Tal1 specifically in the EHT. It is possible that the apoptosis of HE cells associated with the knock-down of β-Tal1 prevented the identification of a prominent role for this isoform of Tal1 in the process of EHT.

Using mESC and a similar differentiation system to ours, Lichtinger et al.^46^ found that before RUNX1 expression, TAL1 and FLI1 are present at promoters of genes linked to EHT in the HE, suggesting that these genes are in a ‘primed’ state. Even though they have also used the mouse ESC system, they did not have the aim of investigating the role of *Tal1* in the specific time-frame of the EHT. They produced results supporting our findings but their work lacked a functional component for the part that concerns *Tal1*.

Despite their prominent role in HSPC formation, we found that 5TFs expression at high levels in *Tal1*^***Δ/Δ***^ endothelial cells was not sufficient for the generation of VE-CAD^+^CD41^+^ cells from these cells, just like their overexpression was not sufficient to generate VE-CAD^+^CD41^+^ cells in *Tal1*^***Δ/Δ***^ hemangioblast cultures. This suggests that *Tal1* might be required in order for the 5TFs to exert their function in the EHT. Indeed, RUNX1, GATA2, ERG, FLI1, TAL1, LMO2 and LYL1 have been proposed to act as a multiprotein complex, and TAL1 is itself an obligate heterodimer the activity of which is largely modulated by protein–protein interactions^10,17,47,48^. It has been shown that TAL1 can recruit transcriptional regulators and chromatin remodeling complexes to its target genes through physical interactions, and similarly it can be directed to its target genes by interactions with other proteins^17,23,47^. Thus, it is possible that TAL1 is required in hemogenic endothelial cells for the assembly of the multiprotein complex that contains the 5TFs, or to recruit transcriptional regulators that mediate essential functions of the 5TFs to this complex. Further experiments investigating the physical and functional interaction between TAL1 and the 5TFs would be of great value to further our understanding of the interplay among these important regulators of blood development.

From a translational viewpoint, our findings suggest that efforts to generate HSPCs in vitro or ex vivo should take in account the important role of *Tal1* in Pre-HPC generation*,* either favoring the use of starting cells which express this hematopoietic regulator, or exploiting alternative strategies to ensure its expression. Endothelial cells are particularly appealing for regenerative medicine purposes^1,2^. By analyzing publicly available datasets of mouse scRNA-seq, we found that the frequency of adult endothelial cells expressing *Tal1* is very low compared to embryonic ones^49^. Based on our results, it appears therefore that any attempt to stimulate such cells to undergo EHT should include an increase in *Tal1* activity.

In conclusion, with our work we have uncovered an unexpected prominent role for *Tal1* in the EHT. Additionally, we have shown that *Tal1*, together with *Lmo2* and *Lyl1*, likely concurs to activate the hematopoietic transcriptional program and suppress the muscle one in cells that transdifferentiate from VSM.

Through our bioinformatic analysis, we also identified the pathways and genes that were differentially expressed and differentially active in cells that acquired a Pre-HPC phenotype upon 5TFs and 8TFs overexpression. Among these, there are likely to be some with regulatory roles in Pre-HSPCs in vivo. Our work therefore also constitutes a resource for the identification and investigation of potential novel regulators of Pre-HSPC formation and the EHT.

## Methods

### Generation of ESC lines and in vitro ESC differentiation into blood and vascular lineages

All doxycycline-inducible ESC lines were generated using the inducible cassette exchange method as described previously^8,50,51^. No animals were used to generate these cell lines. Through gene synthesis (GenScript) and cloning, p2lox-5TFs and p2lox-3TFs plasmids were generated. They were consequently transfected into A2.lox ESCs (kindly provided by Dr. Michael Kyba—University of Minnesota) as described before^8,50^. The functionality of the overexpression constructs of the chosen clones was corroborated by our RNA-seq and diffTF analyses (Fig. 5D, Supplementary Fig. 2, Supplementary file S1).

The production of i5TFs *Tal1*^*Δ/Δ*^ mESCs, i3TFs *Tal1*^*Δ/Δ*^ mESCs cell lines was done using the Crispr/CAS9 technology. Two gRNAs targeting exon-flanking regions of introns 2 and 4 (relative to Tal1- 201 mRNA) were used to generate a deletion in the Tal1 gene (Figs. 4A,B and 6A). The guide RNAs were designed using the CRISPRko function of the sgRNA CRISPick tool (https://portals.broadinstitute.org/gppx/crispick/public). The top candidate of the output list was selected for experiments. An extra G (in orange) before the sequence of the guide was added to the *Tal1* intron 2 guide to improve the efficiency of transcription from the U6 promoter. Sequences complementary to the overhangs created by digestion of the p133-pPB plasmid with BlpI and BstXI were added at both sides of the designed guides (sequences in blue). The guides were purchased from Sigma-Aldrich as sense and complementary antisense oligonucleotides:

*Tal1* intron 2: Sense 5’TTGGACGCACTGAAACCTGAAAAGGTTTAAGAGC3’;

Antisense 5’TTAGCTCTTAAACCTTTTCAGGTTTCAGTGCGTCCAACAAG3’.

*Tal1* intron 4: Sense 5’TTGGATGGTTCTAACCAGTGACAGTTTAAGAGC3’;

Antisense 5’TTAGCTCTTAAACTGTCACTGGTTAGAACCATCCAACAA 3’.

The guides were cloned individually by standard cloning into a p133-pPB plasmid containing an RNA Polymerase III- dependent U6 promoter, a modified gRNA stem loop and a BFP tag kindly provided by Jamie Hackett (EMBL Rome) (Supplementary Fig. 3). The p133-pPB plasmid was next co-transfected with the pX458 plasmid containing a Cas9 nuclease construct and a GFP reporter (pSpCas9-2A-GFP, Addgene ID: 48,138), to disrupt the Tal1 gene in the i5TFs and i3TFs mESC lines. Following transfection, BFP^+^GFP^+^ ESCs were sorted by flow cytometry and seeded at low density on mouse embryonic fibroblasts (MEFs). One week later, ESC single colonies were picked, expanded and genotyped by PCR, using the primers in Supplementary Table 1, and Sanger sequencing.

The maintenance, culture and differentiation of ESCs (EB differentiation, hemangioblast culture and hemogenic culture) were done using the same protocols as described previously^56^ (see also detailed supplementary methods). All ESC lines generated in this study had proper stem cell morphology and were able to give rise to blood, endothelial cells and vascular smooth muscle cells after in vitro differentiation (Figs. 1C and 2C).

### Flow cytometry and cell sorting

Staining was performed as described previously^52^ (see detailed supplementary methods). Cells from EBs, hemangioblast and hemogenic endothelium were stained with different combinations of antibodies (Supplementary Table 2). The 7AAD dye (Invitrogen, A1310) was used to exclude dead cells. FACS analysis was performed using a FACSCanto (Becton Dickinson) and an Attune NxT Flow Cytometer (Thermo Fisher Scientific). Cell sorting was performed using the FACSAria (Becton Dickinson) or by using magnetic sorting (MACS MicroBead Technology, Miltenyi Biotec) and anti-APC MicroBeads (Miltenyi Biotech). Data were later analyzed using FlowJo v10.1r5 (Tree Star, Inc.).

### Nucleic acids extraction, cDNA production and PCR

Genomic DNA (gDNA) extraction was done using DNeasy Blood & Tissue Kit and the QIAmp DNA Micro Kit from Qiagen, RNA extraction with the RNeasy Plus Mini Kit and RNeasy Micro Kit (Qiagen), cDNA production was performed with the RevertAid H Minus RT cDNA Synthesis kit (ThermoFisher Scientific). PCR for genotyping of ESC lines was done using the KAPA2G Robust HotStart ReadyMix (see primer list in Supplementary Table 1). Quantitative PCR (qPCR) was done using the KAPA SYBR FAST ROX low qPCR Master Mix (2X) Kit and a 7500 Real-Time PCR System from Applied Biosystems (see primer list in Supplementary Table 3).

### Assay for transposase-accessible chromatin using sequencing (ATAC-seq) and analysis

Five thousand cells of each population indicated in Fig. 3A were sorted using flow cytometry directly into 1.5 mL Eppendorf tubes in 50µL of PBS 10% FBS. ATAC-seq was performed using the same protocol described previously^28^ (see also detailed supplementary methods). Purified samples were analysed by capillary electrophoresis on a 2100 Bioanalyzer using the Agilent High Sensitivity DNA Kit to verify the quality of the samples and quantify the samples before pooling. Samples were mixed equimolarly into two pools with 10 samples each. The final libraries were analysed by capillary electrophoresis on the Bioanalyzer to verify the quality of the libraries and quantify them before sequencing. The libraries were paired-end sequenced with the Illumina technology on a NextSeq500 (2 × 75 bp read length, mid-output).

The processing of the ATAC-seq data was done using an in-house Snakemake pipeline starting from the raw fastq files produced following from sequencing. The details of the data processing are described in the “ATAC-seq processing” paragraph of the methods section of the paper by Berest, Arnold et al.^30^.

### Bulk RNA-seq and data analysis

Bulk RNA-seq was performed as described by the SmartSeq2 protocol^29^. Briefly, 25 cells from each group (Fig. 3A) were FACS sorted directly into lysis buffer containing 0.2% Triton X-100, oligo-dT primers and dNTP mix, and then snap frozen. Reverse transcription was then performed, followed by pre-amplification for 14 cycles. Nextera libraries were then prepared and sequenced on the Illumina Next Seq sequencer. The processing of the RNA-seq data was performed using the EMBL Galaxy Server (https://galaxy.embl.de/)^53^. FastQC was used to perform quality control on the raw sequencing data. Removal of adaptor sequences (trimming) from paired-end reads was performed with Trim Galore!. FastQC was run again to perform quality control on the trimmed reads. Reads were aligned to the reference mouse genome GRCm38 (UCSC mm10) using the RNA STAR (Spliced Transcripts Alignment to a Reference) Aligner^54^. Filter SAM or BAM was used to filter the BAM files using SAMtools to remove poor quality alignments and retain one alignment per read (Skip alignments with any of these flag bits set: the alignment of this read is not primary, the read fails platform/vendor quality checks, supplementary alignment). bamCoverage (deepTools) was used to generate a coverage bigWig file (bin size in bases: 10, Scaling/Normalization method: 1x). featureCounts was used to count how many reads have mapped to genes (measure gene expression from BAM files). CollectRNASeqMetrics (Picard tools) was used to collect metrics about the alignment of RNA to various functional classes of loci in the genome (http://broadinstitute.github.io/picard/). CollectInsertSizeMetrics (Picard tools) was used to plot the distribution of insert sizes (http://broadinstitute.github.io/picard/).

Differential expression analysis was performed using the DESeq2 package with the R software (version 3.5.1, http://www.R-project.org.) along with R studio (version 0.99.879)^35^.

### diffTF analysis on ATAC-Seq data

diffTF version 1.6 was used with RNA-seq integration, Hocomocov version 11, and cluster.largeAnalysis.json. The following options were set in config.json: "maxCoresPerRule": 20, "nPermutations": 0, "nBootstraps": 1000, "nCGBins": 10, "TFs": "all". The documentation can be found at https://difftf.readthedocs.io/en/v1.6/. The details of the data processing for the diffTF analysis of the ATAC-Seq sequencing data are described in the work by Berest, Arnold et al.^30^.

### String analysis on diffTF results

For String analysis, we used the output of the diffTF analysis corresponding to a TF class stringency of 0.001 and adj. *p*-value < 0.05. We filtered out the TFs that were not expressed according to out RNA-seq analysis on the corresponding cells. For the generation of the interaction networks we used the web tool STRING (https://string-db.org/), and displayed only the TFs that were connected to at least another TFs in the network. For the network generation, we selected the following basic settings: meaning of network edges “confidence”, active interaction sources “Textmining, Experiments, Databases, Co-expression, Neighborhood, Gene Fusion, Co-occurrence”, minimum required interaction score “medium confidence (0.400)”, max number of interactions to show “1st shell -none/query proteins only, 2nd shell -none”. We selected the following advanced settings: network display mode “interactive svg”, display simplifications “hide disconnected nodes in the network”. For the clustering method we selected “MCL clustering” with an inflation parameter of 3.

### Hierarchical clustering of RNA-seq data and GO analysis on the clusters

The hierarchical clustering analysis of the differentially expressed genes (DEGs) of the four dox-treated populations, and the GO analysis on the 10 identified clusters was performed using the R software. All DEGs identified by DESeq2 from the pairwise gene-expression comparisons between untreated controls and dox-treated samples were merged into one omni-comprehensive matrix. Genes in the matrix that were not identified as differentially expressed in one of the conditions were assigned a log2FC value of 0 replacing the “NA” (not available) value. A distance matrix was calculated using the Manhattan distance, and used to perform the clustering into 10 groups using Ward’s minimum variance method to measure the dissimilarity between two clusters of observations. The clustering was performed with the R stats function hclust, and a heatmap was generated with the R function pheatmap.

GO analysis and KEGG^55^ enrichment were performed using the R package clusterProfiler. Emaps for the visualization of GO and KEGG analyses were created using the enrich plot package.

### Motif enrichment analysis with HOMER

Transcription factor motif enrichment analysis of the differentially expressed genes in the i3TFs dox-treated condition was performed with HOMER tools (http://homer.ucsd.edu/homer/)56 as described in the Supplementary methods (“Motif enrichment analysis with HOMER” section).

### Statistical analysis

Each experiment has been performed at least three times. Statistical analysis was done using the R software.

### Protein extraction and quantification

Protein extraction from frozen cell pellets was performed with RIPA buffer (150 mM NaCl, 1% NP-40, 0.5% sodium deoxycholic, 0.1% SDS, 50 mM Tris pH 8.0) containing Protease/Phosphatase Inhibitors (complete MINI EDTA-free by Roche) and treatment with Pierce Universal Nuclease for Cell Lysis (ThermoFisher Scientific), as described in the Supplementary methods (“Protein extraction and quantification” section). Protein concentration was measured using the Pierce BCA Protein Assay Kit (ThermoFisher Scientific).

### Western blot analysis

Protein samples were diluted in RIPA buffer and 4X SDS-Page Loading Buffer (200 mM Tris–HCl pH 6.8, 8% SDS [Sodium dodecyl sulfate], 40% glycerol, 400 mM DTT [dithiothreitol], 0.4% bromophenol blue) and denatured for 5 min at 95 °C before loading onto NuPAGE Novex 12% Bis–Tris Protein gels (ThermoFisher Scientific). Proteins were semi-dry transferred onto a 0.2 µm PVDF membrane (Trans-Blot Turbo Mini 0.2 µm PVDF Transfer Packs from Biorad). Membranes were blocked with TBST (TBS [20 mM Tris-Base, 154 mM NaCl, pH 7.5], 0.1% Tween 20) + 5% milk for 30 min at RT. Primary antibody staining was carried-out overnight at 4 °C, secondary antibody staining was carried out for 30 min at RT. Antibodies were diluted in TBST + 5% milk as follows: anti-HA antibody (Sigma) 1:1000, anti-β-Actin antibody (Sigma) 1:10,000, anti-Mouse HRP-conjugated secondary antibody (GE Healthcare Life Sciences) 1:10,000 (HA-TAL1 staining) and 1:100,000 (B-ACTIN staining). Membranes were washed with 1X PBS + Triton 0.5% + 0.5 M NaCl three times for 5 min and once for 10 min, rinsed once in 1X PBS, and developed using the ECL Prime Western-Blot-System (Merck).

## Supplementary Information

Supplementary Information 1.

Supplementary Information 2.

Supplementary Information 3.

Supplementary Information 4.

Supplementary Information 5.

Supplementary Information 6.

Supplementary Information 7.
