# Supplementary material for: Identifying a novel role for the master regulator Tal1 in the Endothelial to Hematopoietic Transition
Source: Sci Rep. 2022 Oct 10;12:16974. doi: 10.1038/s41598-022-20906-0 (PMC9550822; doi:10.1038/s41598-022-20906-0)
Supplement: Supplementary file 1 — Supplementary Information 1. [file 41598_2022_20906_MOESM1_ESM.pdf]

Supplementary Information for

Identifying a novel role for the master regulator Tal1 in the Endothelial to Hematopoietic Transition

Yasmin Natalia Serina Secanechia, Isabelle Bergiers, Matt Rogon, Christian Arnold, Nicolas Descostes, Stephanie Le, Natalia Lopez Anguita, Kerstin Ganter, Chrysi Kapsali, Lea Bouilleau, Aaron Gut, Auguste Uzuotaite, Ayshan Aliyeva, Judith B. Zaugg and Christophe Lancrin

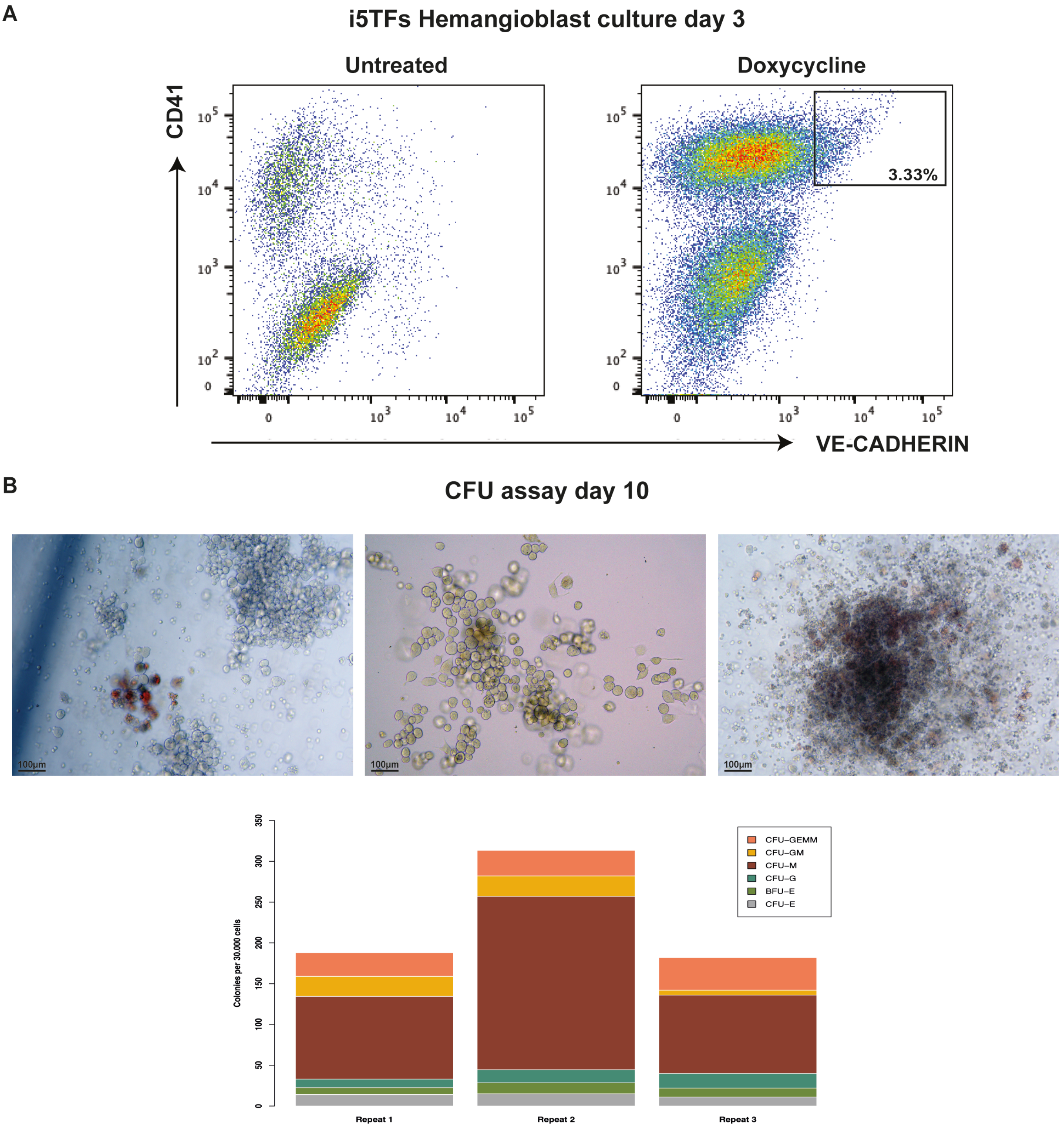

Supplementary Fig. 1: hematopoietic potential of sorted i5TFs VE-CAD$^+$CD41$^+$ cells

A. FACS analysis results showing the expression of VE-CAD and CD41 in day 3 i5TFs haemangioblast cultures. Left panel: untreated cultures. Right panel: cultures treated with doxycycline between days 1 and 2. The square highlights the cells sorted for CFU assay. **B.** Top panel: microscopy images of representative colonies from the CFU assay. Left: BFU-E, CFU-M and sparse granulocytes. Middle: CFU-M. Right: CFU-GEMM. Bottom panel: stacked bar-plot summarizing the results of three independent experiments.

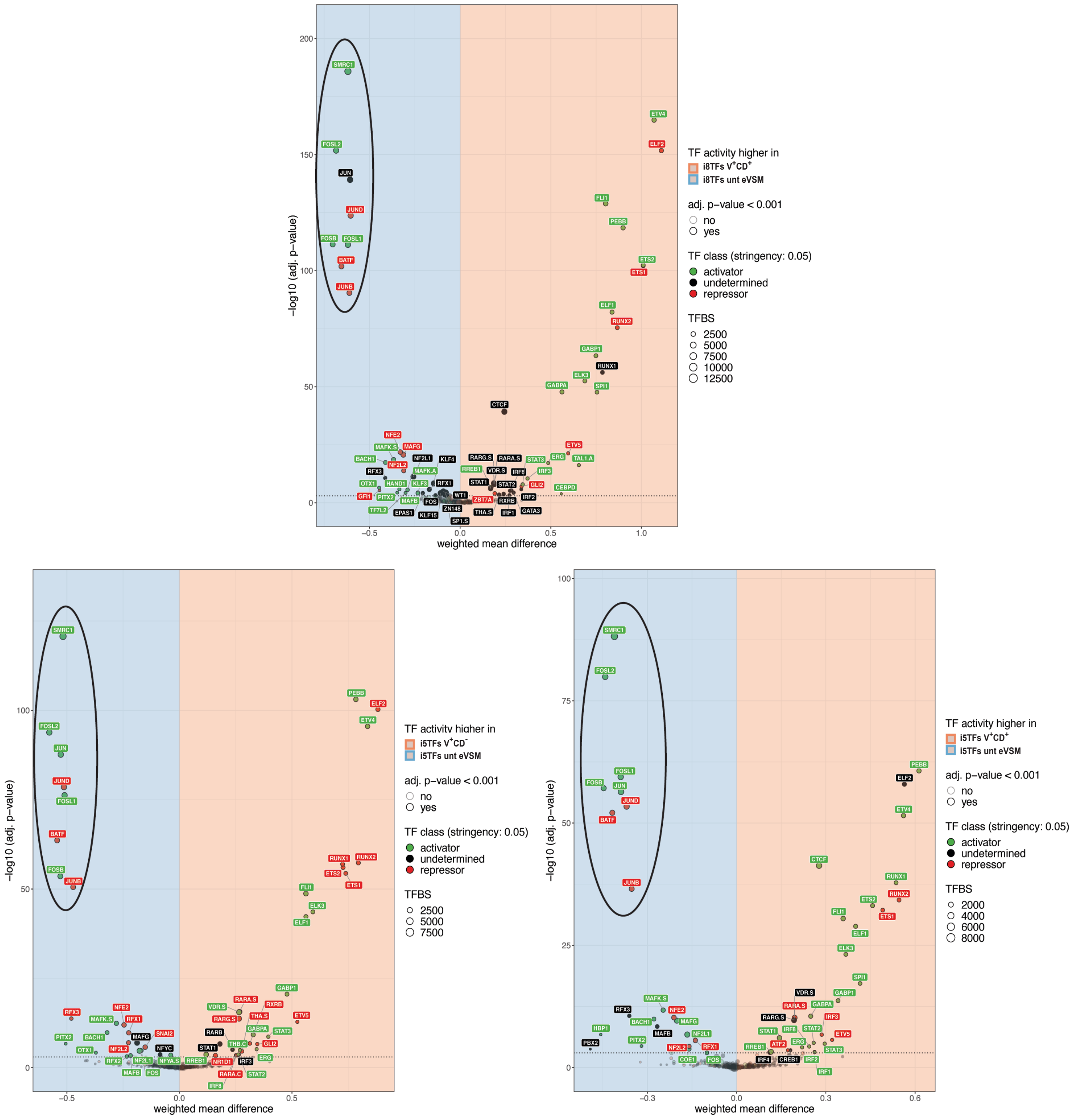

Supplementary Fig. 2: Volcano-plots showing the transcription factors identified as differentially active in dox-treated cells and untreated controls

Volcano-plots showing the transcription factors (TFs) identified by diffTF (Berest, Arnold et al., 2019) as differentially active in untreated cells (blue quadrant) and dox-treated cells (red quadrant). TFs classified as activators are labelled in green, TFs classified as repressors are labelled in red, TFs that couldn't be classified as either are labelled in black. 5% of TFs were classified as activators or repressors (TF class stringency: 0.05) based on the Pearson correlation index. The x axis (weighted mean difference) shows the difference in TF activity between the untreated (unt) and dox-treated (dox) conditions. The y-axis displays the significance of the TFs. The significance threshold is indicated with a dotted line (FDR adjusted P-value < 0.05). Transcription-factor binding sites (TFBS) are indicated as a dot. The size of each dot is proportional to the number of genomic TFBS identified for each TF. VSM-specific transcription factors are highlighted. See also Fig. 3.

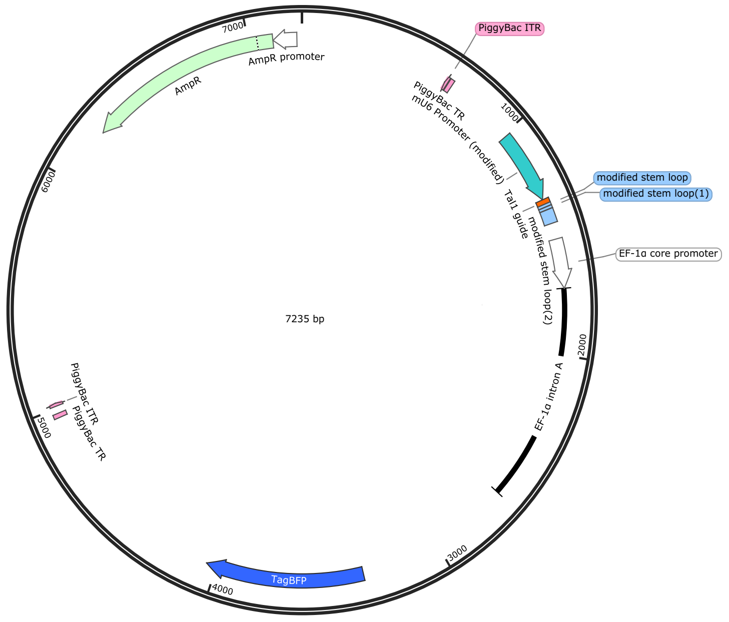

Supplementary Fig. 3: Scheme of the p133-pPB plasmid encoding for the Tal1 gRNA used for the disruption of the *Tal1* gene. See also Fig. 4.

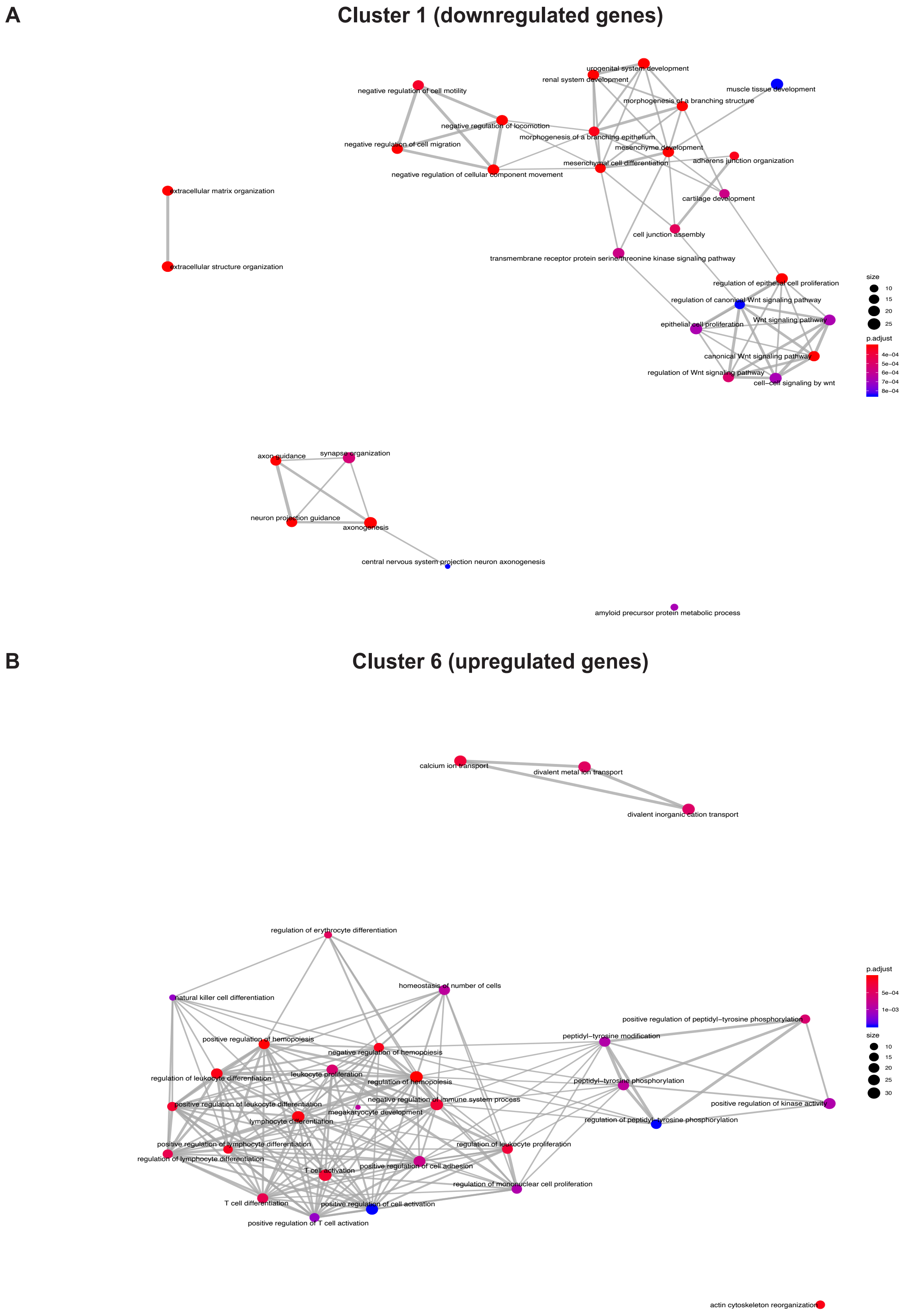

Supplementary Fig. 4: GO terms enriched in clusters one and six

Enrichment maps (EMaps) showing the GO terms enriched in clusters 1 (**A**) and 6 (**B**) identified by the hierarchical clustering of DEGs in i8TFs V^+CD^+, i5TFs V^+CD^+, i5TFs V^+CD^- and i3TFs dox eVSM. Each node (circle) represents a GO term. The size of the nodes is proportional to the number of genes belonging to each GO term, and the color represents the significance. The edges (lines) connect terms with shared genes, so GO terms with mutually overlapping gene sets tend to cluster together. Related to Fig. 5.

A **Cluster 2 (genes downregulated as consequence of 5TFs overexpression)**

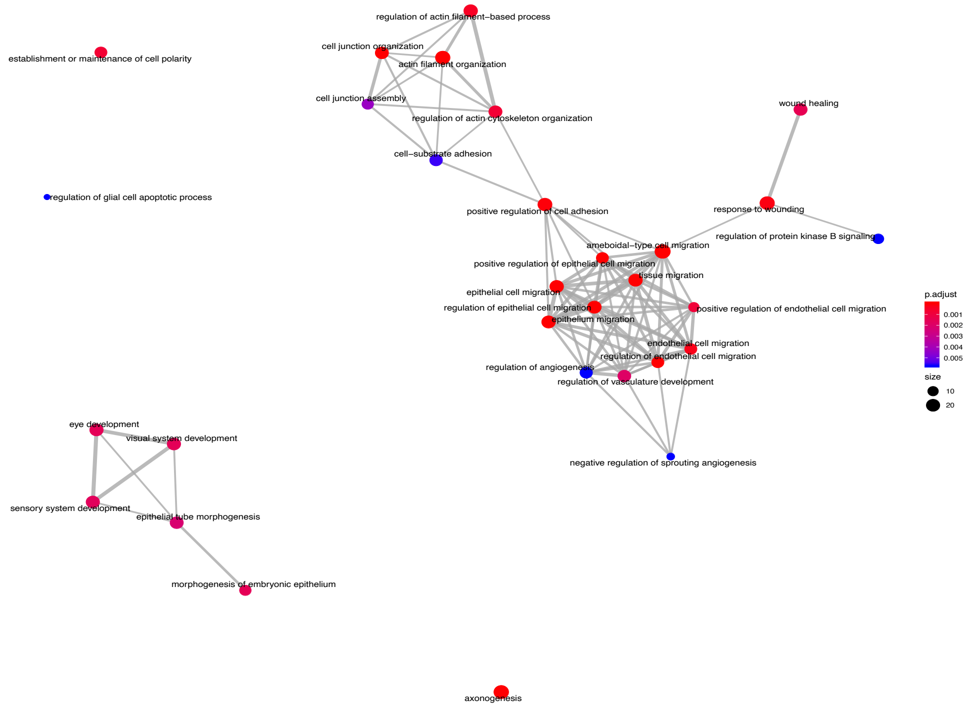

B **Cluster 3 (genes downregulated when the 3TFs were not overexpressed)**

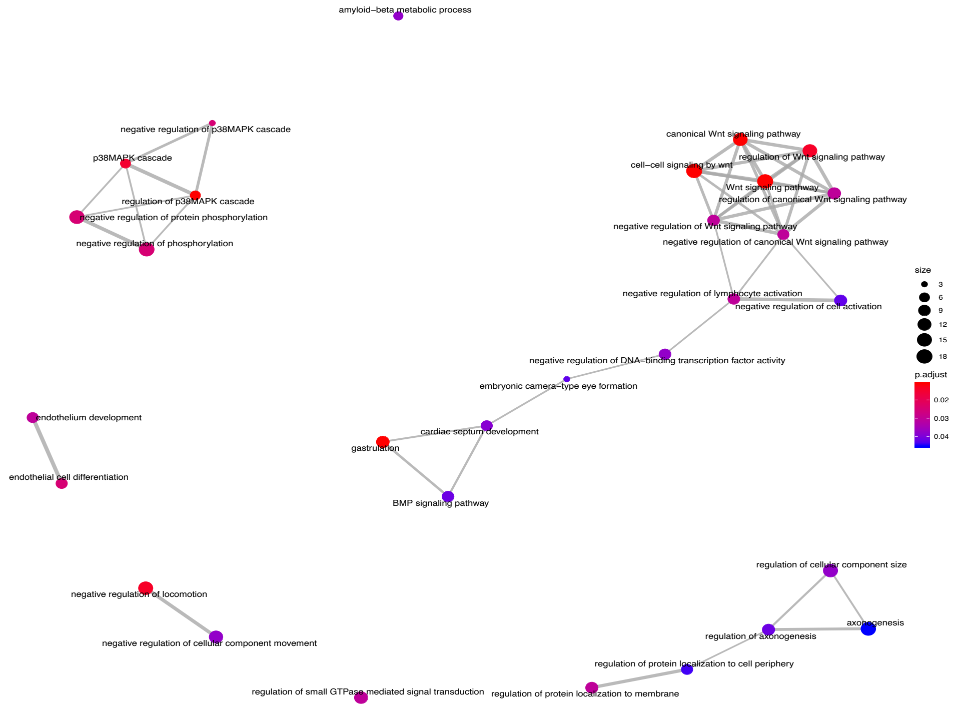

Supplementary Fig. 5: GO terms enriched in clusters two and three

Enrichment maps (EMaps) showing the GO terms enriched in clusters 2 (**A**) and 3 (**B**) identified by the hierarchical clustering of DEGs in i8TFs $V^{+}CD^{+}$, i5TFs $V^{+}CD^{+}$, i5TFs $V^{+}CD^{-}$ and i3TFs dox eVSM. Each node (circle) represents a GO term. The size of the nodes is proportional to the number of genes belonging to each GO term, and the color represents the significance. The edges (lines) connect terms with shared genes, so GO terms with mutually overlapping gene sets tend to cluster together. Related to Fig. 5.

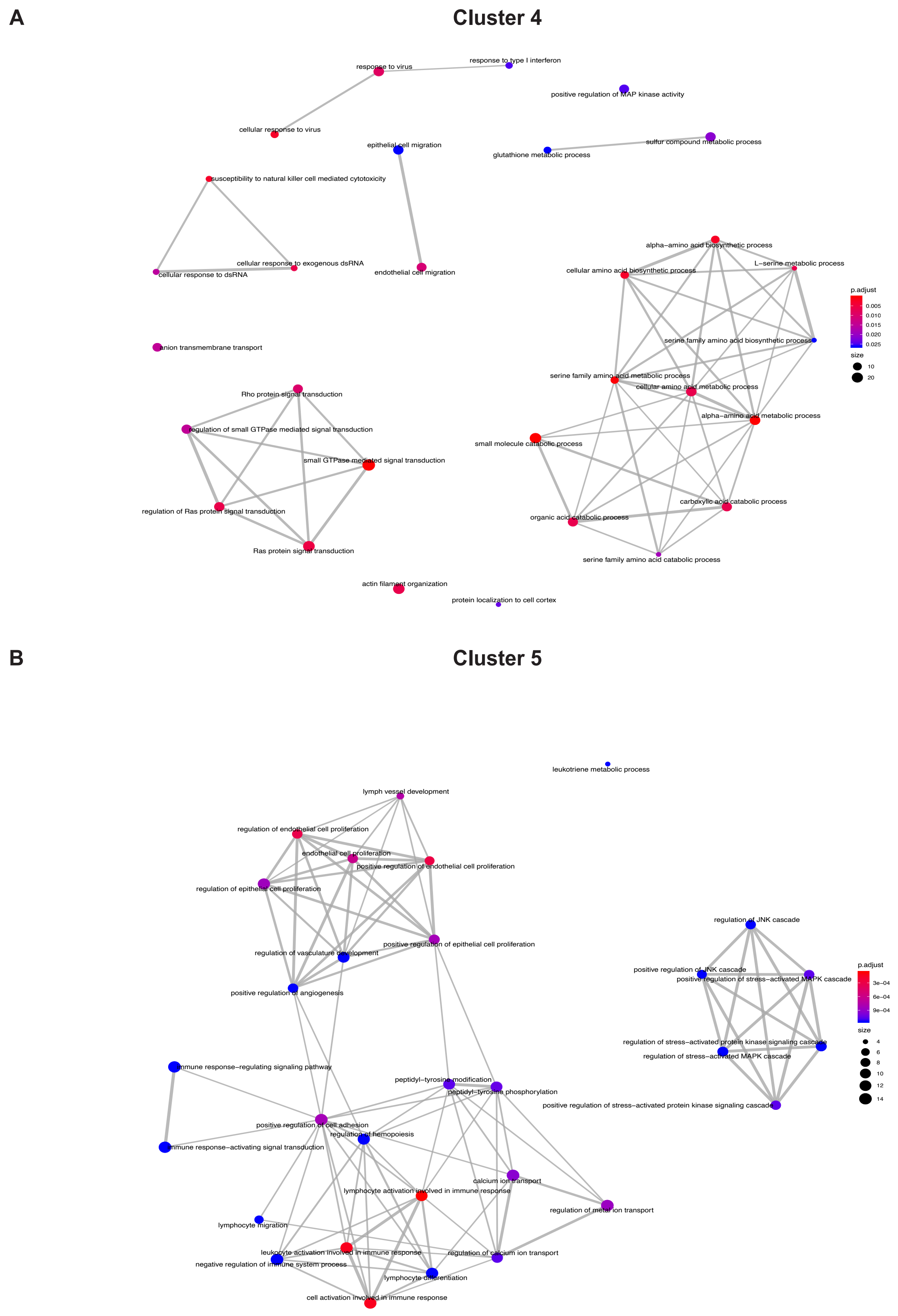

Supplementary Fig. 6: GO terms enriched in clusters four and five

Enrichment maps (EMaps) showing the GO terms enriched in clusters 4 (**A**) and 5 (**B**) identified by the hierarchical clustering of DEGs in i8TFs V$^+$CD$^+$, i5TFs V$^+$CD$^+$, i5TFs V$^+$CD$^-$ and i3TFs dox eVSM. Each node (circle) represents a GO term. The size of the nodes is proportional to the number of genes belonging to each GO term, and the color represents the significance. The edges (lines) connect terms with shared genes, so GO terms with mutually overlapping gene sets tend to cluster together. See also Fig. 5.

A

Cluster 7

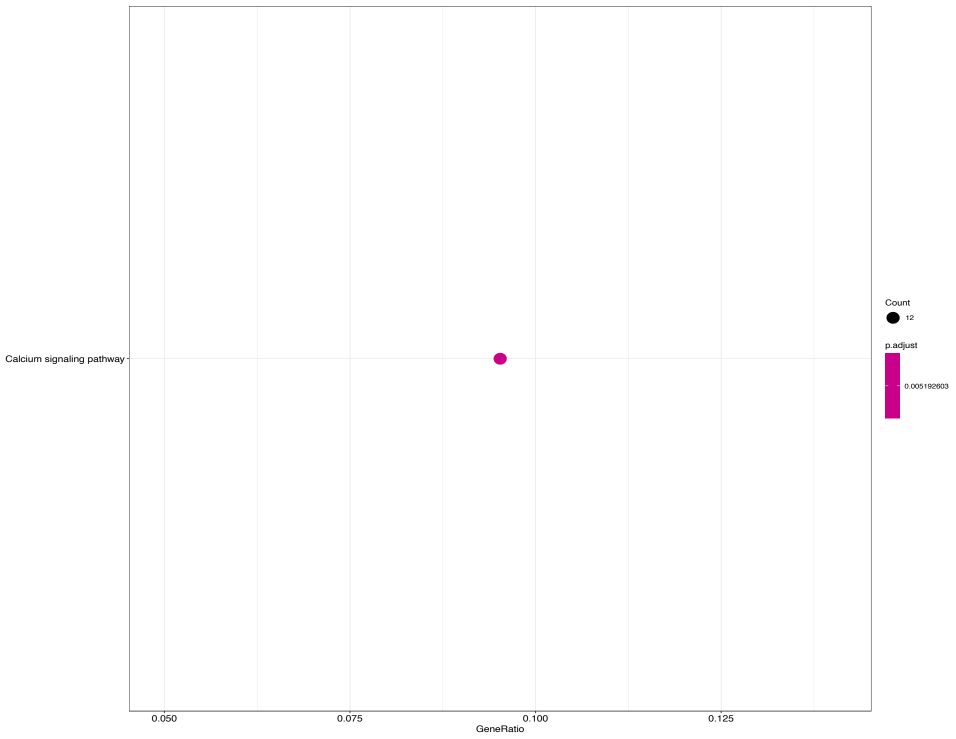

B

Cluster 8

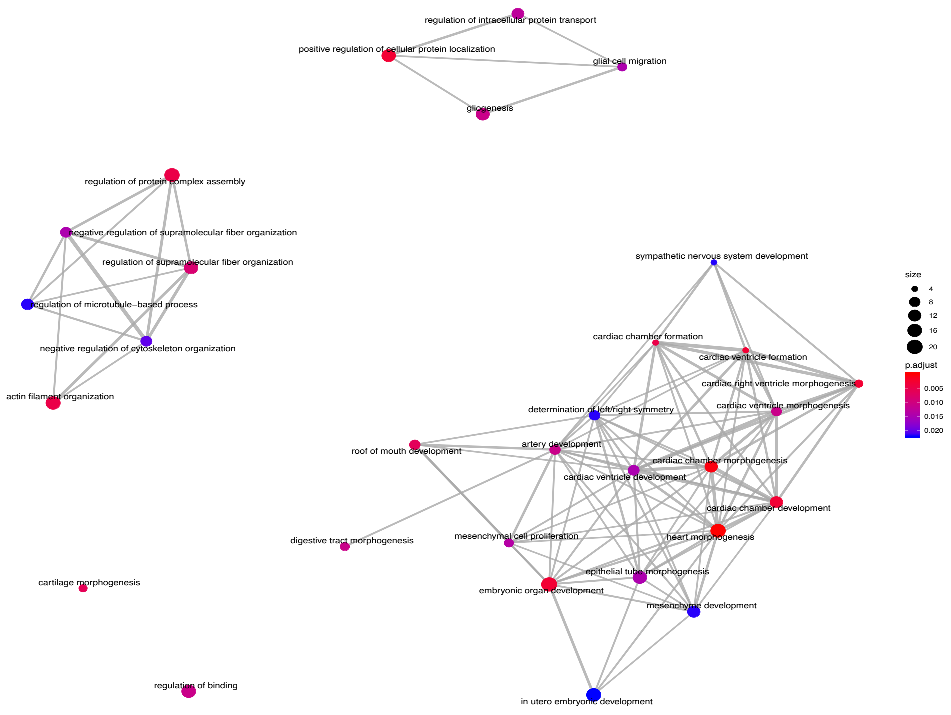

Supplementary Fig. 7: GO terms enriched in clusters seven and eight

Dotplot and Enrichment map (EMap) showing the GO terms enriched in clusters 7 (**A**) and 8 (**B**) identified by the hierarchical clustering of DEGs in i8TFs V+CD+, i5TFs V+CD+, i5TFs V+CD- and i3TFs dox eVSM. Each node (circle) represents a GO term. The size of the nodes is proportional to the number of genes belonging to each GO term, and the color represents the significance. The edges (lines) connect terms with shared genes, so GO terms with mutually overlapping gene sets tend to cluster together. Related to Fig. 5.

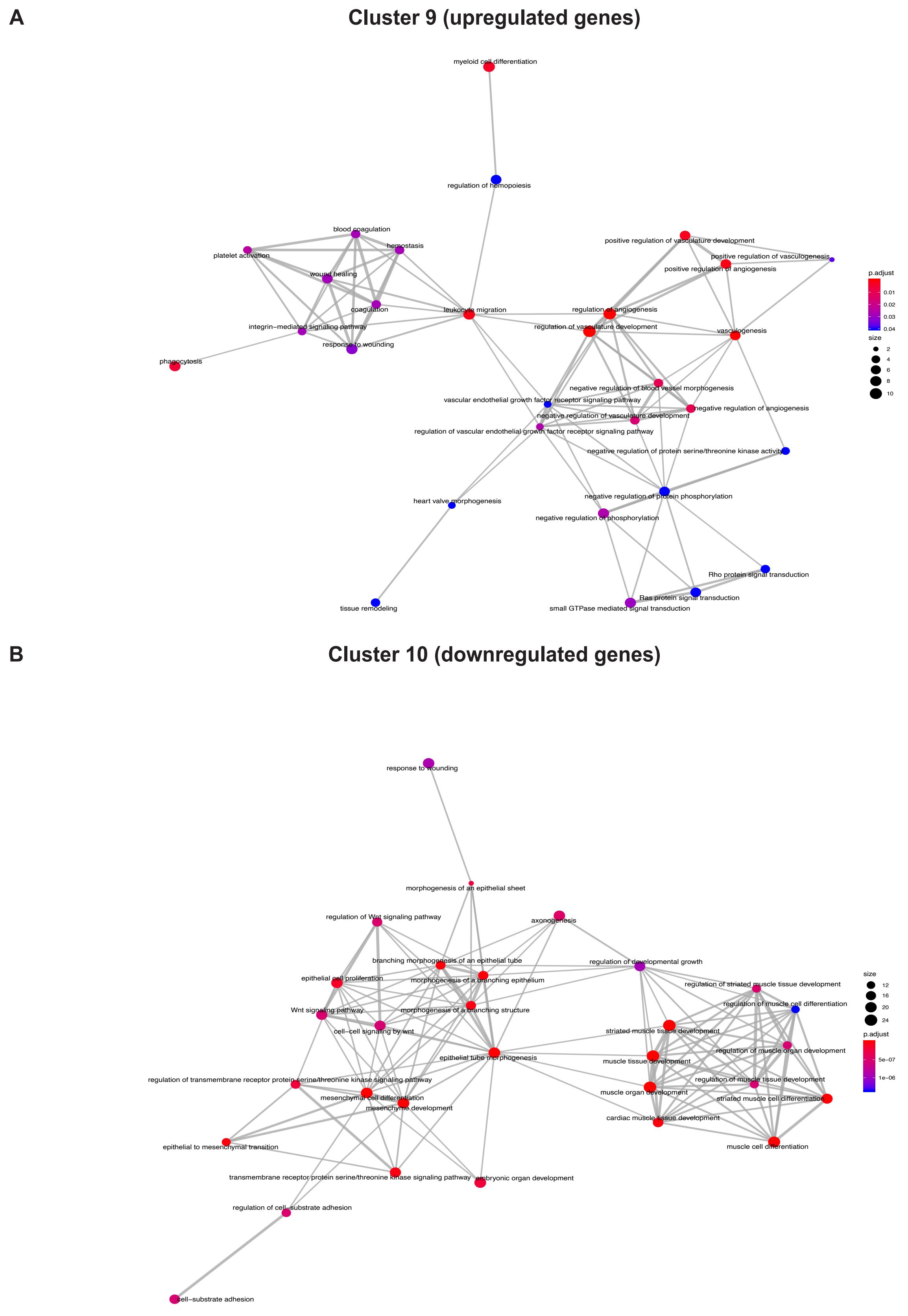

Supplementary Fig. 8: GO terms enriched in clusters seven and eight

Enrichment maps (EMaps) showing the GO terms enriched in clusters 9 (**A**) and 10 (**B**) identified by the hierarchical clustering of DEGs in i8TFs $V^{+}CD^{+}$, i5TFs $V^{+}CD^{+}$, i5TFs $V^{+}CD^{-}$ and i3TFs dox eVSM. Each node (circle) represents a GO term. The size of the nodes is proportional to the number of genes belonging to each GO term, and the color represents the significance. The edges (lines) connect terms with shared genes, so GO terms with mutually overlapping gene sets tend to cluster together. See also Fig. 5.

STRING network analysis

i3TFs unt eVSM

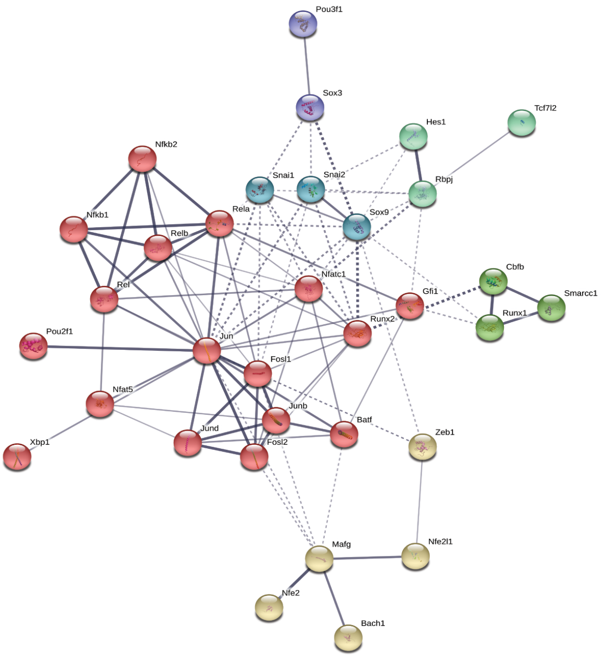

i3TFs dox eVSM

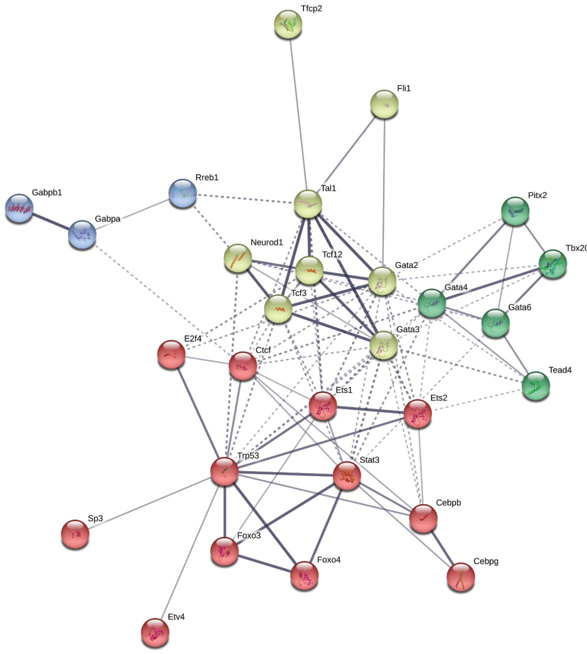

Supplementary Fig. 9: STRING interaction network analysis comparing i3TFs unt eVSM to i3TFs dox eVSM

Interaction networks of TFs predicted by DiffTF to be more active in i3TFs untreated eVSM cells compared to i3TFs dox eVSM cells. Each node represents a TF and each edge represents an interaction (physical or functional, experimentally determine or predicted). Nodes with the same color belong to the same cluster (unsupervised clustering using the Markov model). Continuous edges show interactions among nodes within the same cluster, dotted edges show interactions among nodes of different clusters. The thickness of the edges represents the confidence of the interaction (the strength of data support).

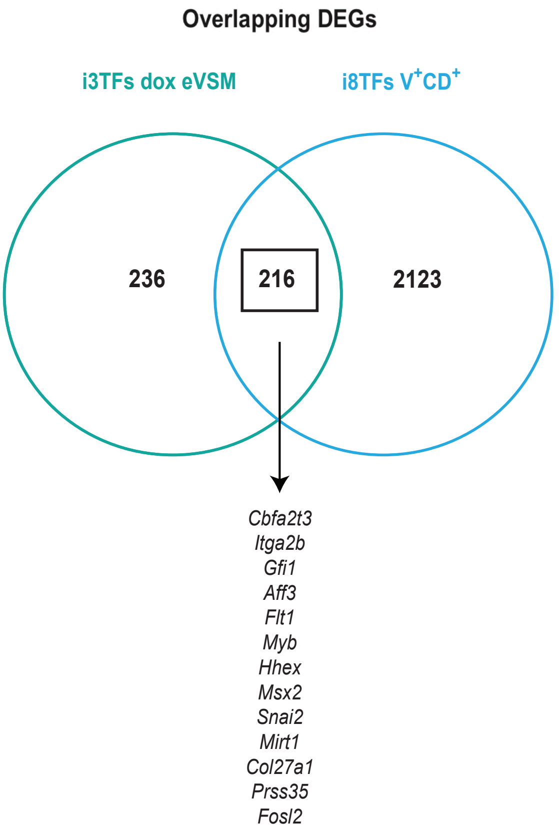

Supplementary Fig. 10: DEG overlap between i3TFs dox eVSM and i8TFs V$^+$CD$^+$

Venn diagrams comparing the DEGs in i3TFs dox eVSM and i8TFs V$^+$CD$^+$. A subset of differentially expressed hematopoietic and muscle-related genes in common between the two conditions is shown. Related to Fig. 5.

Differential expression of *Tal1* target genes in hemangioblast cultures at day 3

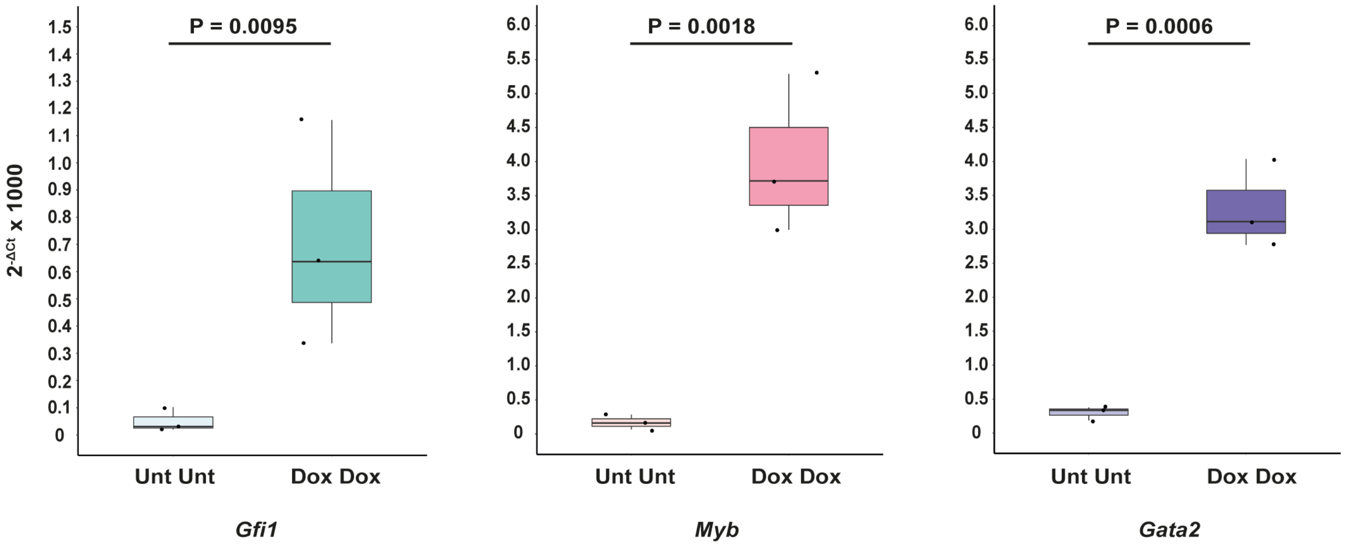

Supplementary Fig. 11: Differential expression of *Tal1* hematopoietic target genes in hemangioblast cultures at day 3

Box plots showing the differential expression of three hematopoietic targets of Tal1 in i3TFs *Tal1*$^{\Delta/\Delta}$ hemangioblast cultures as assessed by qRT-PCR. Unt Unt = untreated; Dox Dox = doxycycline added at EB day 2 and at day 0 of hemangioblast culture. Significance was determined by Analysis of Variance (ANOVA) test. Error bars correspond to standard deviations.

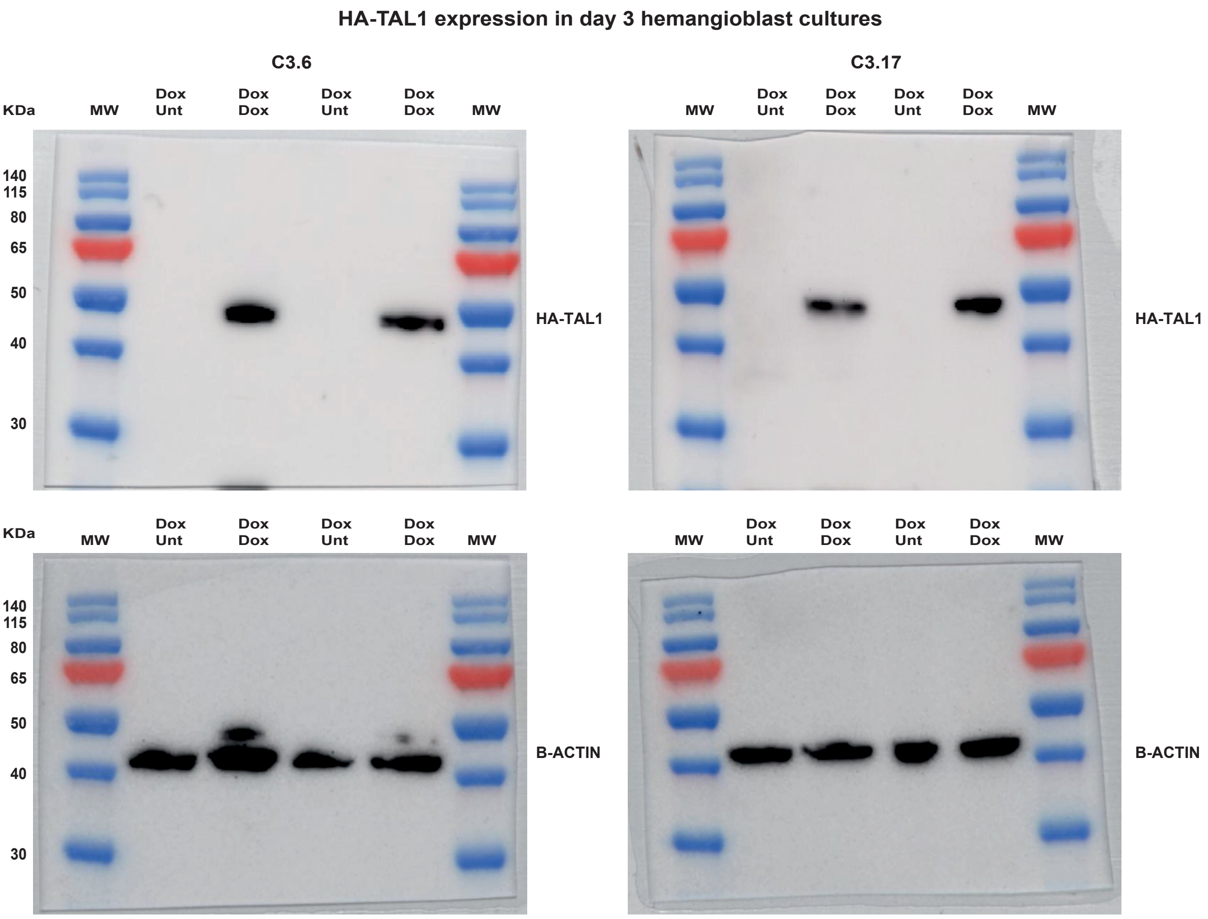

Supplementary Fig. 12: Western blot gels images related to Fig. 7A

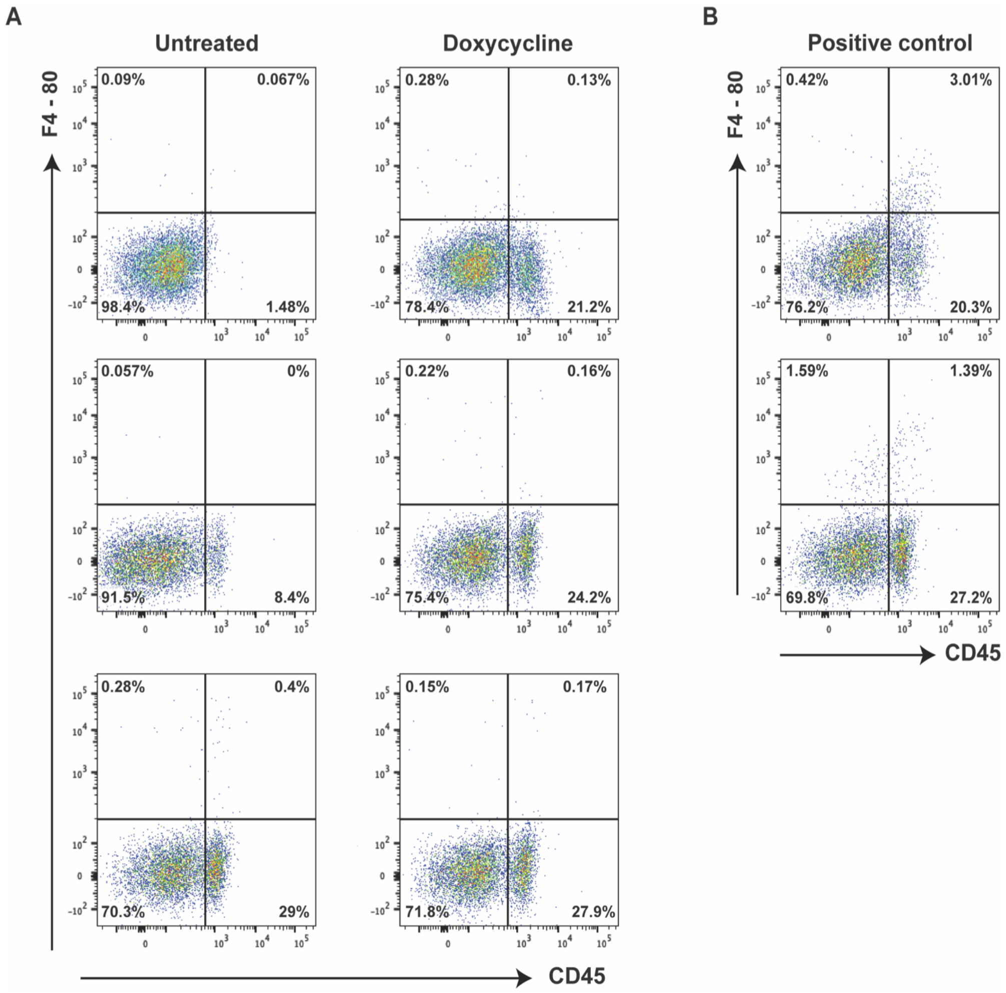

Supplementary Fig. 13: Hematopoietic potential of sorted i3TFs *Tal1*$^{\Delta/\Delta}$ VE-CAD^{+}CD41^{-} cells - hemogenic endothelium cultures

A. FACS analysis results showing the expression of lineage differentiation markers in sorted i3TFs *Tal1*$^{\Delta/\Delta}$ Dox Unt VE-CAD^{+}CD41^{-} cells subjected to hemogenic endothelium culture for 45 hours in the presence or absence of doxycycline, followed by 10 days of hematopoietic differentiation in a liquid big mix in the absence of doxycycline. **B.** Day 3 i3TFs *Tal1*$^{\Delta/\Delta}$ Dox Dox hemangioblast cultures were used as a positive control. CD45: pan-hematopoietic marker; F4-80: macrophage marker. Untreated: no dox added to the hemogenic endothelium culture. Doxycycline: dox added to the hemogenic endothelium culture.

Description of supplementary files

Supplementary file S1: Differentially expressed genes in i8TFs V^+CD^+, i5TFs V^+CD^+, i5TFs V^+CD^- and i3TFs dox eVSM relative to untreated eVSM controls.

Excel file displaying the genes identified by DESEq2 as differentially expressed in i8TFs V^+CD^+, i5TFs V^+CD^+, i5TFs V^+CD^- and i3TFs dox eVSM relative to untreated eVSM controls ($log_2FC > |1|$, Adjusted P-value < 0.05). The first worksheet contains differentially expressed genes in i8TFs V^+CD^+ relative to i8TFs unt eVSM. The second worksheet contains the differentially expressed genes in i5TFs V^+CD^+ compared to i5TFs unt eVSM. The third worksheet contains the differentially expressed genes in i5TFs V^+CD^- compared to i5TFs unt eVSM. The fourth worksheet contains the differentially expressed genes in i3TFs dox eVSM compared to i3TFs unt eVSM. **(Related to Figures 3D, 5A and Supplementary Fig. 4-10).**

Supplementary file S2: Uncropped electrophoresis images.

Word file displaying uncropped images of electrophoresis displayed in Fig.4B and Fig.6A **(Related to Figures 4B and 6A.)**

Supplementary file S3: Gene content of the clusters identified by hierarchical clustering.

Excel file displaying the genes belonging to each of the 10 clusters identified by hierarchical clustering on the differentially expressed genes in i8TFs V^+CD^+, i5TFs V^+CD^+, i5TFs V^+CD^- and i3TFs dox eVSM relative to untreated eVSM controls. **(Related to Figure 5A and Supplementary Fig. 4-8).**

Description of supplementary files

Supplementary file S4: Analysis of the clusters identified by hierarchical clustering.

The documents provide an analysis of the gene clusters identified by hierarchical clustering on the differentially expressed genes in i8TFs V^+CD^+, i5TFs V^+CD^+, i5TFs V^+CD^- and i3TFs dox eVSM relative to untreated eVSM controls. **(Related to Figure 5A and Supplementary Fig. 4-8).**

Supplementary file S5: Gene ontology analysis results of the clusters identified by hierarchical clustering with corresponding genes.

The documents contain the GO terms enriched in each of the clusters identified by hierarchical clustering on the differentially expressed genes in i8TFs V^+CD^+, i5TFs V^+CD^+, i5TFs V^+CD^- and i3TFs dox eVSM relative to untreated eVSM controls, with the corresponding genes. No GO terms were found for cluster 7 therefore we have included a Kegg pathway analysis **(Related to Figure 5A and Supplementary Fig. 4-8).**

Supplementary file S6: Gene expression in Dox Unt Endothelial cells.

Excel file displaying the gene expression values (log_2GeneExpression) from the RNA-Seq analysis of sorted Dox Unt Endothelial cells at day 3 of hemangioblast culture. The expression values derive from the DESEq2 analysis. **(Related to Figure 7C.)**

Supplementary Methods

EXPERIMENTAL MODEL AND SUBJECT DETAILS

CELL LINES

Generation of inducible ESC lines

All doxycycline-inducible ESC lines were generated using the inducible cassette exchange method, the constructs and plasmids described previously [1-3]. No animals were used to generate these cell lines. The generation of the i8TFs mESC line is described in detail in Bergiers et al. For the generation of the i5TFs mESC line, HA-*Tal1*, FLAG-*Lyl1* and V5-*Lmo2* were successively excised from the p2lox-8TFs by classic cloning to generate the p2lox-5TFs used for the generation of the inducible ESC line. For the generation of the i3TFs mESC line, cmyc-*Fli1* and V5-*Erg* were successively excised from the intermediate p2lox construct containing Construct one and Construct two described in Bergiers et al. to generate the p2lox-3TFs used for the generation of the inducible ESC line. The functionality of the overexpression constructs of the chosen clones was corroborated by our RNA-seq and diffTF analyses (Fig.5D, Supplementary Fig. 1, Supplementary file 1).

Generation of cell lines with a deletion in the *Tal1* gene (i5TFs *Tal1*$^{\Delta/\Delta}$ mESCs, i3TFs *Tal1*$^{\Delta/\Delta}$ mESCs)

Two gRNAs targeting exon-flanking regions of introns 2 and 4 (relative to *Tal1*- 201 mRNA) were used to generate a deletion in the *Tal1* gene (Fig. 4A & 4B, Fig. 6A). The intronic sequences were downloaded from the Ensemble genome browser v95 and used as input for the design of gRNAs. GRNAs were designed using the CRISPRko function of the sgRNA Design tool (now CRISPick) in the GPP Web Portal of the Broad Institute (https://portals.broadinstitute.org/gppx/crispick/public). The top candidate of the output list for each gene was selected for experiments. An extra G (in orange) before the sequence of the guide was added to the *Tal1* intron 2 guide to improve the efficiency of transcription from the U6 promoter. Sequences complementary to the overhangs created by digestion of the p133-pPB plasmid with BlpI and BstXI were added at both sides of the designed guides (sequences in blue). The guides were purchased from Sigma-Aldrich as sense and complementary antisense oligonucleotides.

Tal1 intron 2: Sense 5'TTGGACGCACTGAAACCTGAAAAGGTTTAAGAGC3';

Antisense 5'TTAGCTCTTAAACCTTTTCAGGTTTCAGTGCGTCCAACAAG3'.

Tal1 intron 4: Sense 5'TTGGATGGTTCTAACCAGTGACAGTTTAAGAGC3';

Antisense 5'TTAGCTCTTAAACTGTCACTGGTTAGAACCATCCAACAA 3'.

To generate double-stranded oligonucleotides, 10µL of the sense and antisense oligonucleotides (100µM) were mixed with 80µL of 1.2X annealing buffer (10mM Tris pH 7.5-8.0, 60mM NaCl, 1mM EDTA), placed in a heating block at 95°C for 3' and allowed to anneal at RT for at least 30'.

The guides were cloned individually by standard cloning into a p133-pPB plasmid containing an RNA Polymerase III- dependent U6 promoter, a modified gRNA stem loop and a BFP tag kindly provided by Jamie Hackett (EMBL Rome) (Supplementary Fig. 2).

The p133-pPB plasmids encoding for the *Tal1*-gRNAs were co-transfected with the pX458 plasmid containing a *Cas9* nuclease construct and a GFP reporter (pSpCas9-2A-GFP, Addgene ID: 48138), kindly provided by Jamie Hackett from EMBL Rome, to disrupt the *Tal1* gene in the i5TFs and i3TFs mESC lines.

MEFs were plated into gelatin-coated 6-well plates at a confluence of $0.2*10^6$ cells/well. The day before transfection, i5TFs and i3TFs mESCs were seeded on the MEFs at a confluence of $0.3*10^6$ cells/well in 3mL of DMEM-ES. Transient transfection was performed with polyethylenimine (PEI) based on the protocol of Longo and co-workers [4]. Before transfection, the old medium was replaced with 2.7mL of fresh DMEM-ES. For each transfection, 9µg of PEI (kindly provided by the Genetic and viral engineering facility at EMBL-Rome) were diluted in DMEM in a final volume of 150µL. A total of 3µg of plasmid DNA per reaction were diluted in DMEM in a final volume of 150µL. The diluted PEI and DNA were mixed (3:1 ratio of PEI to DNA) and incubated at RT for 30'. The mixture was added to the cells dropwise. 1µg of each plasmid was used per reaction. Transfected cells were harvested after two days, the brightest BFP^+GFP^+ cells were FACS sorted (yields were between 5000 and 7000 cells) and plated in MEF-coated 10cm dishes in 10mL of DMEM-ES. 24 or 48 colonies were picked per cell line one week after sorting, dissociated by treatment with 30µL of TryplE Express for 3' at 37°C and transferred into MEF-coated 96 well plates with 120µL of fresh DMEM-ES. The medium was changed the next morning to remove the TryplE. Growing clones were progressively transferred to MEF-coated 24-well and 6-well plates and frozen in 50% DMEM, 40% FBS and 10% DMSO, or frozen directly in a 96 well plate after splitting by adding 50µL of 2X freezing medium (80% FBS and 20% DMSO) to 40µL of dissociated cells and storing at -80°C. Cells from all clones were expanded in parallel on gelatin-coated plates for genotyping. The deletions in the *Tal1* gene were interrogated by PCR and sanger sequencing (Fig.4B, Fig.6A). The lack of functionality of the *Tal1* gene in the deleted clones was verified in the hemangioblast culture (Fig.4C & 4D, Fig.6D) [5]. Two clones lacking *Tal1* functionality from both transfected mESC lines were selected for experiments (Fig.4B, Fig.6A).

Supplementary Table 1: Primer pairs for genotyping of *Tal1*

Forward primer (5' → 3')	Reverse primer (5' → 3')
TGTCCCACCCTTTTCTTCCA	AGATCACCTCACTGCCTCTG
TGTCCCACCCTTTTCTTCCA	AGGGAGCAAGCCTAAAGTGA
AAGAGTCCAGCTGAGCAGAA	GTCAGAATCAGCCCCTTCCT

Supplementary Table 1 shows the sequences of the primer pairs used to genotype the deletions in the *Tal1* gene.

Identification of cell lines

All cell lines used in this work were mESC lines. All A2lox.Cre-derived mESC lines were generated from the A2lox.Cre mESC line, which was a gift from Dr. Michael Kyba (University of Minnesota) who produced it in his laboratory [2]. All A2lox.Cre-derived mESC lines were generated in our laboratory as described in the section 'Generation of inducible ESC lines'. All ESC lines had proper stem cell morphology and were able to give rise to blood, endothelial cells and vascular smooth muscle cells after in vitro differentiation (Fig.1C, Fig.2C).

Mouse embryonic stem cell (mESC) culture and embryoid bodies (EB) differentiation

All mESCs used in this work were maintained and expanded on a layer of MEFs in DMEM-ES medium composed of DMEM KO (supplemented with 1% Penicillin/Streptomycin, 1% L-glutamine, 1% non-essential amino acids), 15% FBS, 0.024% of LIF (1mg/mL) (produced by the protein expression facility at EMBL, Heidelberg) and 0.24% 50mM 2-Mercaptoethanol. All media were sterile-filtered before use in a Millipore stericup with a 0.22μm filter. Cells were incubated at 37°C with 5% CO 2 and 95% relative humidity.

Before plating of MEFs, culture dishes were gelatin coated by treatment with a solution of 0.1% gelatin in PBS for 20' at room temperature (RT). MEFs were thawed at least 24hours before seeding of mESCs and plated at a confluence of $0.02*10^6$ cells/cm^2 for expansion of mESC stocks and mESC line generation, and $0.017*10^6$ cells/cm^2 for expansion of mESC prior to differentiation. MESCs were typically cultured in 6-well plates. Frozen cells were thawed in DMEM-ES and plated in one well in 4mL of DMEM-ES. Confluent cells were harvested and seeded at a density of $0.02\text{-}0.03*10^6$ cells/cm^2 ($0.2\text{-}0.3*10^6$ cells/well of a 6-well plate) in 2mL of medium for expansion, depending on the growth rate of the cells. The medium was changed daily to mESCs that were to be frozen as a stock. Cells were detached by treatment with TryplE Express (1mL/well of a 6-well plate) and 3-5' of incubation at 37°C.

For differentiation into EBs, mESCs were subjected to two successive passages on gelatin-coated dishes for the removal of MEFs. Confluent cells at the center of the well were harvested following a 2' RT incubation with TryplE Express pre-heated to 37°C, to minimize the detachment and carryover of MEFs. $1.8*10^6$ cells/dish were plated into gelatin-coated 10cm dishes in 10mL of DMDM-ES. Cells were harvested after 24hours and plated for the second gelatin passage in 10mL of IMDM (Iscove's Modified Dulbecco's Medium) -ES medium composed of IMDM (supplemented with 1% Penicillin/Streptomycin, 1% L-glutamine), 15% FBS, 0.024% of LIF (1mg/mL) and 0.24% 50mM 2-Mercaptoethanol. Cells were harvested after 24hours and plated into standard 90mm petri dishes at a confluence of $0.3*10^6$ cells/dish, in 10mL of EB medium composed of IMDM (supplemented with 1% Penicillin/Streptomycin and 1% L-glutamine), 15% FBS, 0.6% Transferrin, 0.03% monothioglycerol (MTG) and 50µg/mL ascorbic acid. Cells were kept in culture for 3-3.5 days and then harvested for BL-CFC sorting. Cultures were checked at day 2 and transferred to new petri dishes if the EBs were attaching to the bottom of the dishes. For the $Tal1^{\Delta/\Delta}$ i3TFs cell line, doxycycline was added to day 2 EBs to a final concentration of 1µg/mL.

Method details

Flow cytometry and cell sorting

Staining was performed as described in Oatley *et al.* [6], or as described in the "Hemogenic endothelium (HE) culture" section. Cells from EBs, hemangioblast, hemogenic endothelium and liquid big mix cultures were stained with different combinations of antibodies. The 7AAD (Invitrogen, A1310) dye was used to exclude dead cells. FACS analysis was performed using a FACSCanto (Becton Dickinson) and an Attune NxT Flow Cytometer (Thermo Fisher Scientific). Cell sorting was performed using the FACSAria (Becton Dickinson) or by using magnetic sorting (MACS MicroBead Technology, Miltenyi Biotec) and anti-APC MicroBeads (Miltenyi Biotech). Data were later analyzed using FlowJo v10.1r5 (Tree Star, Inc.).

Hemangioblast culture

For hemangioblast cultures, MACS sorted FLK1$^+$ BL-CFCs were plated on gelatin-coated plates at a confluence of $0.0105*10^6$ cells/cm^2 (freshly sorted cells) and $0.0168*10^6$ cells/cm^2 (frozen cells) in a hemangioblast medium containing IMDM (supplemented with 1% Penicillin/Streptomycin and 1% L-glutamine), 10% FBS, 0.6% human transferrin, 0.3% MTG, 50µg/mL ascorbic acid, 0.05% VEGF (10µg/mL) and 0.1% IL-6 (10µg/mL). 15% D4T supernatant was also added to the medium in experiments performed with the i3TFs, i5TFs, i8TFs mESCs (Fig. 1-3). This is the supernatant of

endothelial D4T cells cultured in IMDM media with 10% FBS and 30mg of endothelial growth supplement. BL-CFCs were cultured in hemangioblast medium for up to 3 days, and were harvested at different time-points for downstream analyses. Doxycycline was added to the cells to a final concentration of 1µg/mL.

Hemogenic endothelium (HE) culture

FLK1$^-$CD41$^-$ VSM cells from day 1 hemangioblast cultures and VE-CAD$^+$CD41$^-$ endothelial cells from day 3 hemangioblast cultures were FACS-sorted and plated in gelatin-coated dishes at a density of 0.0095*10^6 cells/cm^2. Cells were cultured up to 2 days in a hematopoiesis-promoting HE medium containing IMDM (supplemented with 1% Penicillin/Streptomycin and 1% L-glutamine), 10% FBS, 1% L-glutamine, 0.6% human transferrin, 0.3% MTG, 50µg/mL ascorbic acid, 0.024% of LIF (1mg/mL), 0.5% SCF (10µg/mL), 0.1% oncostatin M (10µg/mL) and 0.01% FGF (10µg/mL). Doxycycline was added to the FLK1$^-$CD41$^-$ VSM cells to a final concentration of 10µg/mL. Doxycycline was added to the VE-CAD$^+$CD41$^-$ endothelial cells to a final concentration of 1µg/mL.

For FACS and flow cytometry analysis, cells were stained with the antibodies shown in Supplementary table 2. Unstained, single stained and fluorescence minus one (FMO) controls were prepared and analyzed during each experiment. Compensation beads (BD Biosciences) were used for single-stained controls. Cells heat-shocked at 60°C for 5' were used for the 7-AAD single-stained control. 0.1 or 0.05*10^6 cells were typically used for controls. 5000 cells were used for controls in experiments where the cell numbers were limiting (analysis of the hemogenic endothelial cultures from sorted VE-CAD$^+$CD41$^-$ cells). Separate sets of Unstained and FMO controls were typically prepared for untreated and dox-treated conditions. When the cell numbers were limiting (analysis of the hemogenic endothelial cultures from sorted VE-CAD$^+$CD41$^-$ cells) cells from both conditions were pooled together for the controls. 0.1*10^6 cells were typically stained for flow cytometry analysis, and at least 30.000 events per sample were acquired. When cell numbers were limiting, all the remaining cells after aliquoting of the controls were stained, and the maximum number of events possible per sample was acquired.

Cells were stained in a final volume of 50 or 60µL of staining mix (FACS buffer with antibodies). For FACS sorting, all the cells remaining after aliquoting of the controls were stained, and the maximum possible number of cells was sorted. Cells for sorting were stained in 200µL of staining mix per 10^6 cells.

For staining, 2X or 3X (depending on the number of antibodies used for staining) antibody solutions were prepared, and equal volumes of each were mixed to obtain a final 1X staining mix. FMO controls were stained by adding directly 25µL of 2X antibody solution or 20µL of 3X antibody

solutions with an equal volume of FACS buffer to the tubes. For staining, cells were resuspended in staining mix and incubated for 10' at RT, in the dark shaking at 500 RPM. Cells were washed after staining with 1mL of FACS buffer to remove unbound antibodies, and resuspended in FACS buffer with 7-AAD (100X) diluted 1:100 (volume/volume ratio). Unstained controls were resuspended in 50 or 60µL of FACS buffer.

Compensation beads were stained by adding 1 drop of negative beads (CompBead Negative negative Control), 1 drop of positive beads (CompBead Anti-Rat/Hamster Ig, k beads) and 50 or 60µL of staining mix into a tube and incubating in the dark at RT for 5' or in ice for 20'.

Supplementary Table 2: Antibodies used for flow cytometry analysis and FACS sorting

Antigen	Fluorochrome	Working dilution	Clone	Reference
CD309 (FLK1)	APC	1:300	Avas12a1	eBioscience; 17-5821-81
CD144 (VE-CADHERIN)	eFluor-660	1:200	eBioBV13	eBioscience; 50-1441-82
CD41a (CD41)	PE	1:400	MWReg30	eBioscience; 12-0411-82
CD117 (C-KIT)	BV421	1:200	2B8	BD Biosciences; 562609
CD45	BV605	1:100	30-F11	BD Biosciences; 563053
F4/80	PE	1:200	BM8	eBioscience; 12-4801-80

Liquid big mix culture

The supernatant of HE cultures (see "Hemogenic Endothelium culture") was collected after 45 hours, centrifuged at 1200 RPM for 5 minutes, washed in 1X PBS, centrifuged at 1200 RPM for 5 minutes, resuspended in liquid big mix and re-plated in the original wells. Adherent cells were washed with 1X PBS and maintained in liquid big mix until the supernatant was transferred back. Cells were cultured for 10 days and then harvested for FACS analysis. Liquid big mix composition: IMDM (supplemented with 1% Penicillin/Streptomycin and 1% L-glutamine), 15% FBS, 1% L-glutamine, 0.6% human transferrin, 0.3% monothioglycerol (MTG), 50µg/mL ascorbic acid, 0.1% SCF (10µg/mL), 0.1% IL-3 (25µg/mL), 0.1% GM-CSF (25µg/mL), 0.04% IL-11 (12.5µg/mL), 0.2%

erythropoietin (EPO) (10 μg /mL), 0.1% IL-6 (10μg/mL), 0.2% thrombopoietin (TPO) (12.5μg/mL), 0.05% M-CSF (10μg/mL).

Colony Forming Unit (CFU) assay in MethoCult

i5TFs day 3 hemangioblast cultures treated with doxycycline between days 1 and 2 were harvested with TryplE Express and stained with antibodies against VE-CADHERIN, CD41 and C-KIT (see "Hemogenic endothelium (HE) culture"). VE-$CAD^{+}CD41^{+}$ cells were FACS sorted into FACS tubed containing IMDM + 10% FBS, centrifuged for 5 minutes at 1200 RPM, resuspended in IMDM + 2% FBS and seeded onto 35mm culture dishes in Methocult complete medium (MethoCult GF M3434 - Stem Cell Technologies) according to manufacturer's instructions at a concentration of 3×10^5 cells per dish. Colonies were quantified after 7 days.

Genomic DNA (gDNA) extraction

gDNA extraction for genotyping was performed on snap-frozen culture-derived cells using the DNeasy Blood & Tissue Kit and the QIAmp DNA Micro Kit from Qiagen, according to manufacturer's instructions. DNA samples were stored at -20°C.

Genotyping of cell lines

PCRs for the genotyping of cell lines were performed using the KAPA2G Robust HotStart ReadyMix according to manufacturer's instructions using an annealing temperature of 60°C. The primers used for each gene were described previously. Reactions were set-up mixing 6.25μL of 2X KAPA2G master mix, 0.625μL of 10μM forward primer, 0.625μL of 10μM reverse primer, 100ng of gDNA and nuclease-free H_2O to a final volume of 12.5μL.

RNA extraction

RNA extraction was performed on snap-frozen culture-derived cells using the RNeasy Plus Mini Kit and RNeasy Micro Kit according to manufacturer's instructions, based on the amount of starting material. Prior to RNA extraction, cells were homogenized using a syringe and 20-gauge needle or the QIAshredder homogenizer. The RNA was stored at -80°C.

Reverse transcription and cDNA production

cDNA production was performed using the RevertAid H Minus RT cDNA Synthesis kit (ThermoFisher Scientific) according to manufacturer's instructions, starting from 200ng of RNA. The reaction was set-up mixing 1μL of random hexamer primer, 4μL of 5X reaction buffer, 1μL of RiboLock Rnase Inhibitor (20U/μL), 2μL of dNTP mix 10mM, 1μl of RevertAid H Minus M-MuLV

Reverse Transcriptase (200 U/µL) and 200ng of RNA with nuclease-free H_2O for molecular biology in a final volume of 20µL. The reaction was incubated in a thermocycler for 5' at 25°C, 60' at 42°C and 70°C for 5'. The cDNA was stored at -20°C.

Quantitative PCR

qPCR reactions were performed with a 7500 Real-Time PCR System from Applied Biosystems, using the KAPA SYBR FAST ROX low qPCR Master Mix (2X) Kit. The reactions were set-up mixing 5µL of KAPA SYBR FAST qPCR Master Mix (2X) with 0.4µL of 10µM forward primer, 0.4µL of 10µM reverse primer, 2µL of cDNA and 3.2µL nuclease-free H_2O in a final volume of 10µL.

Reactions were cycled as follows: hold at 50°C for 2', enzyme activation at 95°C for 10', 40 cycles of exponential amplification at 95°C for 15'', 60°C for 1' (signal acquisition was performed at this stage), 1 cycle of amplification for melting curve analysis at 95°C for 30'', 60°C for 1', 95°C for 30'' (signal acquisition was performed between the two last stages), and 60°C for 15''.

The mouse house-keeping gene *Ppia* (*Peptidylprolyl Isomerase A*) was used as an internal control for normalization in all qPCR reactions.

The primer pairs used for qPCR are listed below:

Supplementary Table 3: Primer pairs used for qPCR

Gene	Forward primer (5' → 3')	Reverse primer (5' → 3')
Ppia	CGCGTCTCCTTCGAGCTGTTTG	TGTAAAGTCACCACCCTGGCACAT
Tal1	TATAGCCTTAGCCAGCCGC	TTGGTGTGAGGACCATCAGA

Assay for Transposase-Accessible Chromatin using sequencing (ATAC-seq)

ATAC-seq was performed based on the protocol described by Buenrostro and coworkers [7]. 5000 cells were sorted directly into 1.5mL Eppendorf tubes in 50µL of PBS 10% FBS. Cells were centrifuged at 500 x g for 5' at 4°C, the supernatant was carefully removed and cells were gently resuspended in 100µL of cold (pre-cooled at 4°C) lysis buffer (10mM Tris-HCl, pH 7.4, 10mM NaCl, 3mM $MgCl_2$, 0.1% NP40/IGEPAL CA-630, 0.1% Tween-20). Cells were immediately centrifuged at 500 x g for 10' at 4°C. The supernatant was removed and cells were washed once with 50µL of cold 1 x PBS and centrifuged at 500 x g for 5' at 4°C. The supernatant was carefully removed, the nuclei were resuspended in 50µL of transposition mix composed of 25µL of TD (2X reaction buffer), 2.5µL of TDE (Nextera Tn5 Transposase) and 22.5µL of molecular grade nuclease-free H_2O, and incubated at 37°C for 30'. The transposed DNA was purified using the Qiagen MinElute PCR Purification Kit following manufacturer's instructions and eluted in 10µL of Elution Buffer (10mM

Tris buffer, pH 8). The purified product was frozen and stored at -20°C for later amplification of the transposed regions. The entire volume (10µL) of eluted DNA was used for PCR amplification with 2.5µL of Custom Nextera PCR primer 1 (containing barcodes), 2.5µL of Custom Nextera PCR primer 2 (containing barcodes), 25µL of NEBNext High-Fidelity 2X PCR Master Mix and 10µL of molecular-grade nuclease-free H2O for a final 50µL reaction. The reaction was thermal cycled as follows: 1 cycle at 72°C for 5', 1 cycle at 98°C for 30'', 5 cycles of exponential amplification at 98°C for 10'', 63°C for 30'' and 72°C for 1'. 5µL of the reaction were used for qPCR to determine the remaining number of amplification cycles for each sample. For qPCR, 5µL of amplified DNA were mixed with 0.5µL of PCR Primer Cocktail, 0.09µL of 100X SYBR green I, 5µL of NEBNext High-Fidelity 2X PCR Master Mix and 4.41µL of molecular-grade nuclease-free H_2O for a final 15µL reaction. The qPCR was cycled as follows: 1 cycle at 98°C for 30'', 20 cycles of exponential amplification at 98°C for 10'', 63°C for 30'' and 72°C for 1'. The additional number of cycles needed per sample was calculated by plotting the linear Rn value versus the number of cycles and determining the cycle number that corresponds to 1/3 (one third) of the maximum fluorescence intensity.

The remaining 45µL of the PCR reaction were amplified for the number of cycles calculated with the qPCR (1 cycle at 72°C for 5', 1 cycle at 98°C for 30'', N cycles of exponential amplification at 98°C for 10'', 63°C for 30'' and 72°C for 1') and stored at -20°C. The amplification products were purified using SPRI beads (Beckman Coulter) according to the manufacturer's instructions, using a bead-to-DNA ratio of 1.1X, and eluted in 22µL of TE Buffer (TrisHCl 10mM, pH 8).

Purified samples were analyzed by capillary electrophoresis on a 2100 Bioanalyzer using the Agilent High Sensitivity DNA Kit to verify the quality of the samples and quantify the samples before pooling. Samples were mixed equimolarly into two pools with 10 samples each. The final libraries were analyzed by capillary electrophoresis on the Bioanalyzer to verify the quality of the libraries and quantify them before sequencing. The libraries were paired-end sequenced with the Illumina technology on a NextSeq500 (2 x 75bp read length, mid-output) at the Genomics Core Facility at EMBL Heidelberg.

RNA sequencing (RNA-seq)

RNA-Seq was performed according to the SmartSeq2 protocol described by Picelli and coworkers[8]. 25 cells were FACS sorted directly into 0.2mL safe-lock microtubes in 4µL of a freshly-prepared solution containing 2µL of lysis buffer (1µL of RNase Inhibitor 2U/µL with 19µL of a 0.2% (vol/vol) Triton X-100 solution), 1µL of oligo-dT30VN primer 10µM and 1µL of dNTP mix 10mM. The tubes were quickly vortexed, spun down, immediately snap-frozen in dry ice and stored at -80°C for later processing. The steps of cDNA conversion, pre-amplification, purification

with magnetic beads and quality control were performed at the Genomics Core Facility at EMBL Heidelberg or in house. The quality of the cDNA samples was assessed by capillary electrophoresis on a 2100 Bioanalyzer using the Agilent High Sensitivity DNA Kit. The sequencing library preparation and sequencing were performed at the Genomics Core Facility at EMBL Heidelberg. Libraries containing 24 samples each were paired-end sequenced with the Illumina technology on a NextSeq500 (2 x 75bp read length, high-output).

Protein extraction and quantification

Frozen cell pellets from day 3 hemangioblast cultures were thawed in ice and resuspended in 20µL of PBS per 1-5x10^6 cells. 80µL per 1-5x10^6 cells of ice-cold RIPA buffer (150 mM NaCl, 1% NP-40, 0.5% sodium deoxycholic, 0.1% SDS, 50 mM Tris pH 8.0) containing Protease/Phosphatase Inhibitors (cOmplete MINI EDTA-free Protease Inhibitor Cocktail by Merck) were added and the mixture was incubated in ice for 5 minutes. 1µL of Pierce Universal Nuclease for Cell Lysis (ThermoFisher Scientific) per 100µL was added to the mixture followed by 5 minutes of incubation at 37°C. The mixture was centrifuged at maximum speed for 2 minutes at 4°C and the supernatant was transferred to a new tube. Protein concentration was measured using the Pierce BCA Protein Assay Kit (ThermoFisher Scientific).

Western Blot Analysis

Equal amounts of protein samples were diluted in RIPA buffer and 4X SDS-Page Loading Buffer (200mM Tris-HCl pH 6.8, 8% SDS [Sodium dodecyl sulfate], 40% glycerol, 400mM DTT [dithiothreitol], 0.4% bromophenol blue), denatured for 5 minutes at 95°C, spun down and loaded onto NuPAGE Novex 12% Bis-Tris Protein gels (ThermoFisher Scientific). Migration was performed using the XCell SureLock Mini-Cell Electrophoresis System from Invitrogen in NuPAGE MOPS SDS running buffer (ThermoFisher Scientific) with NuPAGE Antioxidant (ThermoFisher Scientific), prepared according to manufacturer's instructions. Semi-dry transfer onto a 0.2µm PVDF membrane (Trans-Blot Turbo Mini 0.2 µm PVDF Transfer Packs from Biorad) was performed using the Trans-Blot Turbo Transfer System from Biorad. Membranes were stained with Ponceau S solution (Sigma) to control the transfer efficiency and protein amounts, washed with TBST (TBS [20 mM Tris-Base, 154 mM NaCl, pH 7.5], 0.1% Tween 20) and blocked with TBST +5% milk for 30 minutes at RT. For primary antibody staining membranes were incubated overnight at 4°C with primary antibodies diluted in TBST +5% milk as follows: anti-HA antibody (Sigma) diluted 1:1000 and anti-β-Actin antibody (Sigma- used as a loading control) diluted 1:10000. For secondary antibody staining membranes were incubated for 30 minutes at RT with an anti-Mouse HRP-conjugated secondary

antibody (GE Healthcare Life Sciences) diluted 1:10000 (HA-Tal1 staining) and 1:100000 (β-Actin staining) in TBST +5% milk.

Following antibody incubation, the membranes were washed with 1X PBS + Triton 0.5% + 0.5M NaCl three times for 5 minutes and once for 10 minutes and rinsed once in 1X PBS.

The membranes were developed using the ECL Prime Western-Blot-System (Merck).

Quantification and statistical analysis

Flow cytometry analysis

All flow cytometry experiments were independently repeated at least three times. The box plots were generated using the R software. One-way ANOVA (Analysis of variance) was performed on the cell frequencies. Error bars correspond to standard deviations.

RNA-seq data analysis

The processing of the RNA-seq data was performed using the EMBL Galaxy Server (https://galaxy.embl.de/) [9]. FastQC was used to perform quality control on the raw sequencing data. Removal of adaptor sequences (trimming) from paired-end reads was performed with Trim Galore!. FastQC was run again to perform quality control on the trimmed reads. Reads were aligned to the reference mouse genome GRCm38 (UCSC mm10) using the RNA STAR (Spliced Transcripts Alignment to a Reference) Aligner[10](Dobin et al., 2013). Filter SAM or BAM was used to filter the BAM files using SAMtools to remove poor quality alignments and retain one alignment per read (Skip alignments with any of these flag bits set: the alignment of this read is not primary, the read fails platform/vendor quality checks, supplementary alignment)[11] (Li et al., 2009). bamCoverage (deepTools) was used to generate a coverage bigWig file (bin size in bases: 10, Scaling/Normalization method: 1x) [12](Ramírez et al., 2016). featureCounts was used to count how many reads have mapped to genes (measure gene expression from BAM files)[13] (Liao et al., 2014). CollectRNASeqMetrics (Picard tools) was used to collect metrics about the alignment of RNA to various functional classes of loci in the genome (http://broadinstitute.github.io/picard/). CollectInsertSizeMetrics (Picard tools) was used to plot the distribution of insert sizes (http://broadinstitute.github.io/picard/).

Differential expression analysis was performed using the DESeq2 package with the R software (version 3.5.1, http://www.R-project.org.) along with R studio (version 0.99.879).

Hierarchical clustering of RNA-seq data and GO analysis on the clusters

The hierarchical clustering analysis of the differentially expressed genes (DEGs) of the four dox-treated populations, and the GO analysis on the 10 identified clusters was performed using the R software. All DEGs identified by DESeq2 from the pairwise gene-expression comparisons between untreated controls and dox-treated samples were merged into one omni-comprehensive matrix. Genes in the matrix that were not identified as differentially expressed in one of the conditions were assigned a log_2FC value of 0 replacing the "NA" (not available) value. A distance matrix was calculated using the Manhattan distance, and used to perform the clustering into 10 groups using Ward's minimum variance method to measure the dissimilarity between two clusters of observations [14]. The clustering was performed with the R stats function hclust, and a heatmap was generated with the R function pheatmap.

GO analysis and KEGG enrichment were performed using the R package clusterProfiler. Emaps for the visualization of GO and KEGG analyses were created using the enrich plot package.

Motif enrichment analysis with HOMER

Transcription factor motif enrichment analysis of the differentially expressed genes in the i3TFs dox-treated condition was performed with HOMER tools (http://homer.ucsd.edu/homer/) [15]. Motif search was done at +/- 2000bp of the annotated gene transcription start sites using all expressed RNAs in i3TFs untreated and dox-treated cells with an rlog > 2 as a background (Supplementary file S1). The analysis was performed searching for an enrichment of known motifs. Statistical significance is indicated in the corresponding figure.

Data and software availability

Software

All software is freely or commercially available and is listed in the Methods description and product reference table.

Supplementary Table 4: Reagents and commercial kits

**Product or resource**	**Supplier**	**Reference number**
**Antibodies**		
Anti-APC MicroBeads	Miltenyi Biotec	130-090-855
Anti-CD144 eFluor-660, eBioBV13	eBioscience	50-1441-82

Anti-Mouse CD309 APC, Avas12a1	eBioscience	17-5821-81
Anti-Mouse CD41 PE, MWReg30	eBioscience	12-0411-81
Anti-Mouse CD45 BV605, 30-F11	BD Biosciences	563053
Anti-Mouse F4/80 PE, BM8	eBioscience	12-4801-80
**Chemicals and commercial assays**		
100X SYBR Green I	Invitrogen	S7563
2-mercaptoethanol	GIBCO	31350–010
Ascorbic acid	Sigma-Aldrich	A4544
Betaine	Sigma-Aldrich	61962
CompBead Anti-Rat and Anti-Hamster Ig κ /Negative Control Compensation Particles	BD Biosciences	552845
cOmplete MINI EDTA-free Protease Inhibitor Cocktail	Merck	1183617001
D4T supernatant	EMBL-Rome	N/A
DMEM KnockOut (KO)	GIBCO	10829–018
Doxycycline	Sigma-Aldrich	D9891
DTT	Invitrogen	18064-014
EPO	R and D	959-ME-010
Fetal bovine serum (FBS)	GIBCO	10270106
Gelatin	BDH	440454B
GM-CSF	R and D	415 ML-010
Human FGF basic	R and D	233-FB-025
IGEPAL CA 630	Sigma-Aldrich	I8896
KAPA SYBR FAST qPCR Master Mix (2X) Kit	KAPABIOSYSTEMS	KR0389_S
KAPA2G Robust HotStart ReadyMix PCR Kit	KAPABIOSYSTEMS	KR0381_S
IL-11 (recombinant murine)	Preprotech	220-11
IL-3 (recombinant murine)	Preprotech	213-13
IL-6 (recombinant murine)	Preprotech	216-16
IMDM	Lonza	BE12-726F
IMDM	Sigma	I3390
L-glutamine	GIBCO	25030–024
LIF	EMBL Heidelberg	N/A
LightCycler 480 SYBR Green I Master	Roche	04707516001

Magnesium chloride ($MgCl_2$)	Sigma-Aldrich	M8266
MCSF (recombinant murine)	Preprotech	315-02
MEM Non-Essential Amino Acids Solution	GIBCO	11140–035
MinElute PCR Purification Kit	QIAGEN	28004
Monothioglycerol (MTG)	Sigma-Aldrich	M6145
NEBNext High-Fidelity 2X PCR Master Mix	New England Biolabs	M0541
Nextera Index Kit	Illumina	FC-121-1011
NuPAGE Antioxidant	ThermoFisher Scientific	NP0005
NuPAGE MOPS SDS running buffer 20X	ThermoFisher Scientific	NP0001
NuPAGE Novex 12% Bis-Tris Protein gels	ThermoFisher Scientific	NP0341
Oncostatin M	R and D	495-MO
PageRuler Prestained Protein Ladder, 10 to 180kDa	ThermoFisher Scientific	26616
Penicillin-streptomycin	GIBCO	15140–122
Pierce BCA Protein Assay Kit	ThermoFisher Scientific	23225
Pierce Universal Nuclease for Cell Lysis 5kU	ThermoFisher Scientific	88701
Recombinant RNase inhibitor	Clontech	2313A
RNeasy Micro Kit	QIAGEN	74004
RNeasy Plus Mini Kit	QIAGEN	74134
SCF (recombinant murine)	Preprotech	250-03
SPRISelect	Beckman Coulter	B23318
Superscript II reverse transcriptase	Invitrogen	18064-014
TD (2X reaction buffer from Nextera kit)	Illumina	FC-121-1030
TDE (Nextera Tn5 transposase from Nextera kit)	Illumina	FC-121-1030
TPO (recombinant murine)	Preprotech	315-14
Trans-Blot Turbo Mini 0.2 µm PVDF Transfer Packs	Biorad	1704156

Transferrin	Roche (Italy)	10652202001
Triton X-100	Sigma-Aldrich	T9284
TrypLE express	GIBCO	12605–036
TWEEN 20	Sigma-Aldrich	P1379
VEGF (recombinant human)	Preprotech	100-20

Supplementary Methods References

1. Bergiers, I., Andrews, T., Vargel Bölükbaşı, Ö., Buness, A., Janosz, E., Lopez-Anguita, N., Ganter, K., Kosim, K., Celen, C., Itır Perçin, G., et al. (2018). Single-cell transcriptomics reveals a new dynamical function of transcription factors during embryonic hematopoiesis. eLife *7*, R106.

2. Iacovino, M., Bosnakovski, D., Fey, H., Rux, D., Bajwa, G., Mahen, E., Mitanoska, A., Xu, Z., and Kyba, M. (2011). Inducible Cassette Exchange: A Rapid and Efficient System Enabling Conditional Gene Expression in Embryonic Stem and Primary Cells. Stem Cells *29*, 1580–1588.

3. Vargel, Ö., Zhang, Y., Kosim, K., Ganter, K., Foehr, S., Mardenborough, Y., Shvartsman, M., Enright, A.J., Krijgsveld, J., and Lancrin, C. (2016). Activation of the TGFβ pathway impairs endothelial to haematopoietic transition. Scientific Reports *6*, 21518.

4. Longo, P.A., Kavran, J.M., Kim, M.-S., and Leahy, D.J. (2013). Transient mammalian cell transfection with polyethylenimine (PEI). Methods Enzymol *529*, 227–240.

5. Lancrin, C., Sroczynska, P., Stephenson, C., Allen, T., Kouskoff, V., and Lacaud, G. (2009). The haemangioblast generates haematopoietic cells through a haemogenic endothelium stage. Nature *457*, 892–895.

6. Oatley, M., Bölükbaşı, Ö.V., Svensson, V., Shvartsman, M., Ganter, K., Zirngibl, K., Pavlovich, P.V., Milchevskaya, V., Foteva, V., Natarajan, K.N., et al. (2020). Single-cell transcriptomics identifies CD44 as a marker and regulator of endothelial to haematopoietic transition. Nature Communications *11*, 586–18.

7. Buenrostro, J.D., Wu, B., Chang, H.Y., and Greenleaf, W.J. (2015). ATAC-seq: A Method for Assaying Chromatin Accessibility Genome-Wide. Curr Protoc Mol Biol *109*, 21.29.1–21.29.9.

8. Picelli, S., Faridani, O.R., Björklund, A.K., Winberg, G., Sagasser, S., and Sandberg, R. (2014). Full-length RNA-seq from single cells using Smart-seq2. Nature Protocols *9*, 171–181.

9. Afgan, E., Baker, D., Batut, B., van den Beek, M., Bouvier, D., Čech, M., Chilton, J., Clements, D., Coraor, N., Grüning, B.A., et al. (2018). The Galaxy platform for accessible, reproducible and collaborative biomedical analyses: 2018 update. Nucleic Acids Research *46*, W537–W544.

10. Dobin, A., Davis, C.A., Schlesinger, F., Drenkow, J., Zaleski, C., Jha, S., Batut, P., Chaisson, M., and Gingeras, T.R. (2013). STAR: ultrafast universal RNA-seq aligner. Bioinformatics *29*, 15–21.

11. Li, H., Handsaker, B., Wysoker, A., Fennell, T., Ruan, J., Homer, N., Marth, G., Abecasis, G., Durbin, R., 1000 Genome Project Data Processing Subgroup (2009). The Sequence Alignment/Map format and SAMtools. Bioinformatics *25*, 2078–2079.

12. Ramírez, F., Ryan, D.P., Grüning, B., Bhardwaj, V., Kilpert, F., Richter, A.S., Heyne, S., Dündar, F., and Manke, T. (2016). deepTools2: a next generation web server for deep-sequencing data analysis. Nucleic Acids Research *44*, W160–W165.

13. Liao, Y., Smyth, G.K., and Shi, W. (2014). featureCounts: an efficient general purpose program for assigning sequence reads to genomic features. Bioinformatics *30*, 923–930.

14. Murtagh, F., and Legendre, P. (2014). Ward's Hierarchical Agglomerative Clustering Method: Which Algorithms Implement Ward's Criterion? J Classif *31*, 274–295.

15. Heinz S, Benner C, Spann N, Bertolino E et al (2010). Simple Combinations of Lineage-Determining Transcription Factors Prime cis-Regulatory Elements Required for Macrophage and B Cell Identities. Mol Cell *38*, 576-589.
