# Supplementary material for: Identifying a novel role for the master regulator Tal1 in the Endothelial to Hematopoietic Transition
Source: Sci Rep. 2022 Oct 10;12:16974. doi: 10.1038/s41598-022-20906-0 (PMC9550822; doi:10.1038/s41598-022-20906-0)
Supplement: Supplementary file 3 — Supplementary Information 3. [file 41598_2022_20906_MOESM3_ESM.docx]

**Uncropped electrophoresis image from Fig. 4B**

**Uncropped electrophoresis images from Fig. 6A**
