# Supplementary material for: Identifying a novel role for the master regulator Tal1 in the Endothelial to Hematopoietic Transition
Source: Sci Rep. 2022 Oct 10;12:16974. doi: 10.1038/s41598-022-20906-0 (PMC9550822; doi:10.1038/s41598-022-20906-0)
Supplement: Supplementary file 4 — Supplementary Information 4. [file 41598_2022_20906_MOESM4_ESM.docx]

Cluster 1 contained mostly genes that were downregulated in i8TFs V+CD+ but not in i3TFs dox eVSM, i5TFs V+CD+ and i5TFs V+CD-, suggesting that these genes were downregulated as a consequence of the combined action of the 5TFs with the 3TFs.

Cluster 2 contained mostly genes that were clearly downregulated as a consequence of 5TF overexpression, with seemingly no contribution from the 3TFs.

Cluster 3 contained mostly genes that appeared to be strongly downregulated when the 3TFs were not overexpressed, suggesting that the regulation of gene expression by the 5TFs was modulated by the presence of the 3TFs.

Genes in clusters 4 were upregulated in i5TFs V+CD-, with the majority being upregulated also in i5TFs Pre-HSPCs and a big portion also in i8TFs V+CD+.

Genes in clusters 5 were upregulated in i5TFs V+CD-, i5TFs V+CD+ and i8TFs V+CD+.

Custer 6 contained genes that were differentially expressed upon 3TFs and 8TFs overexpression.

Clusters 7 and 8 contained mostly genes which were upregulated and downregulated, respectively, in cells that acquired a Pre-HPC phenotype, mostly upon overexpression of the 5TFs compared to the 8TFs. Some of the genes in cluster 8 were also differentially expressed upon 3TFs overexpression.

Clusters 9 and 10 contained genes that were overexpressed and downregulated, respectively, upon 5TF and 8TFs overexpression, with some of these genes also being differentially expressed upon 3TFs overexpression.

Interestingly, some of the DEGs in clusters 6 appeared to be downregulated when only the 3TFs were overexpressed, while they were upregulated when all 8TFs were overexpressed. Similarly, a few of the genes in cluster 10 were upregulated in i3TFs dox eVSM but downregulated in i8TFs V+CD+. These findings seem to suggest that the interaction with the 5TFs modulated the activator versus repressive activity of the 3TFs.
